# Comprehensive molecular biomarker identification in breast cancer brain metastases

**DOI:** 10.1186/s12967-017-1370-x

**Published:** 2017-12-29

**Authors:** Hans-Juergen Schulten, Mohammed Bangash, Sajjad Karim, Ashraf Dallol, Deema Hussein, Adnan Merdad, Fatma K. Al-Thoubaity, Jaudah Al-Maghrabi, Awatif Jamal, Fahad Al-Ghamdi, Hani Choudhry, Saleh S. Baeesa, Adeel G. Chaudhary, Mohammed H. Al-Qahtani

**Affiliations:** 1 Center of Excellence in Genomic Medicine Research, King Abdulaziz University, Jeddah, Saudi Arabia; 2 Division of Neurosurgery, Department of Surgery, King Abdulaziz University Hospital, Jeddah, Saudi Arabia; 3 King Fahad Medical Research Center, King Abdulaziz University, Jeddah, Saudi Arabia; 4 Department of Surgery, Faculty of Medicine, King Abdulaziz University, Jeddah, Kingdom of Saudi Arabia; 5 Department of Pathology, Faculty of Medicine, King Abdulaziz University Hospital, Jeddah, Saudi Arabia; 6 Department of Pathology, King Faisal Specialist Hospital and Research Center, Jeddah, Saudi Arabia; 7 Biochemistry Department, Faculty of Science, King Abdulaziz University, Jeddah, Saudi Arabia

**Keywords:** Breast cancer brain metastases, Whole transcript array profiling, Pathway and network analysis, snoRNAs, Copy number variations, Whole exome sequencing

## Abstract

**Background:**

Breast cancer brain metastases (BCBM) develop in about 20–30% of breast cancer (BC) patients. BCBM are associated with dismal prognosis not at least due to lack of valuable molecular therapeutic targets. The aim of the study was to identify new molecular biomarkers and targets in BCBM by using complementary state-of-the-art techniques.

**Methods:**

We compared array expression profiles of three BCBM with 16 non-brain metastatic BC and 16 primary brain tumors (prBT) using a false discovery rate (FDR) *p* < 0.05 and fold change (FC) > 2. Biofunctional analysis was conducted on the differentially expressed probe sets. High-density arrays were employed to detect copy number variations (CNVs) and whole exome sequencing (WES) with paired-end reads of 150 bp was utilized to detect gene mutations in the three BCBM.

**Results:**

The top 370 probe sets that were differentially expressed between BCBM and both BC and prBT were in the majority comparably overexpressed in BCBM and included, e.g. the coding genes *BCL3*, *BNIP3*, *BNIP3P1*, *BRIP1*, *CASP14*, *CDC25A*, *DMBT1*, *IDH2*, *E2F1*, *MYCN*, *RAD51*, *RAD54L*, and *VDR*. A number of small nucleolar RNAs (snoRNAs) were comparably overexpressed in BCBM and included *SNORA1*, *SNORA2A*, *SNORA9*, *SNORA10*, *SNORA22*, *SNORA24*, *SNORA30*, *SNORA37*, *SNORA38*, *SNORA52*, *SNORA71A*, *SNORA71B*, *SNORA71C*, *SNORD13P2*, *SNORD15A*, *SNORD34*, *SNORD35A*, *SNORD41*, *SNORD53*, and *SCARNA22*. The top canonical pathway was entitled, role of BRCA1 in DNA damage response. Network analysis revealed key nodes as Akt, ERK1/2, NFkB, and Ras in a predicted activation stage. Downregulated genes in a data set that was shared between BCBM and prBT comprised, e.g. BC cell line invasion markers JUN, MMP3, TFF1, and HAS2. Important cancer genes affected by CNVs included *TP53*, *BRCA1*, *BRCA2*, *ERBB2*, *IDH1*, and *IDH2*. WES detected numerous mutations, some of which affecting BC associated genes as *CDH1*, *HEPACAM*, and *LOXHD1*.

**Conclusions:**

Using complementary molecular genetic techniques, this study identified shared and unshared molecular events in three highly aberrant BCBM emphasizing the challenge to detect new molecular biomarkers and targets with translational implications. Among new findings with the capacity to gain clinical relevance is the detection of overexpressed snoRNAs known to regulate some critical cellular functions as ribosome biogenesis.

**Electronic supplementary material:**

The online version of this article (10.1186/s12967-017-1370-x) contains supplementary material, which is available to authorized users.

## Background

It is estimated that 20–30% of all breast cancer (BC) develop BC brain metastases (BCBM) [1]. Recent studies indicate that BCBM is highest in ERBB2 (HER2) and triple negative BC (TNBC) with an incidence of 20–50% [2–5]. The high incidence of BCBM in ERBB2 tumors possibly could be attributed to ERBB2 targeted treatment that leads to an initially increased survival [6, 7]. Mean time between primary BC and BCBM is approximately 35 months and main associated variables are tumor size and lymph node metastasis [8, 9]. Approximately 30% of BC patients reveal to have a BCBM at autopsy [10, 11]. The majority of patients receive a multimodality therapy approach that may include treatment with an anti**-**Human ERBB2 therapeutic antibody in ERBB2 positive tumors and hormonal therapy in ER and/or PR positive tumors [9]. *ERBB2* amplifications and mutations are frequently found in BC and corresponding BCBM [12]. Targeted therapy options for triple negative BCBM, which harbor frequently *BRCA1* and *BRCA2* aberrations, are currently not available in clinical practice. To improve treatment of BCBM, a number of advanced therapy trials and new targeted therapy options are emerging [1, 13–15]. Molecular targets include e.g. ERBB2, EGFR, VEGFR, PARP, and the mTOR and CDK-4/6 pathways.

The brain metastatic process is a multistep sequence involving migration, intravasation, circulation, arrest, extravasation, and settlement/invasion of the brain microenvironment [5, 16, 17]. Especially, the blood brain barrier (BBB) is highly selective for both tumor cells and drug therapeutics to enter the brain microenvironment. Consistent with this, it has been demonstrated that brain metastatic lesions have a monoclonal or predominantly monoclonal origin [5, 18–21]. This implicates that a brain metastasis shares common aberrations with the metastatic ancestor cell while subsequent evolving aberrations may only be present in brain metastatic subclones. A support for this may be the fact that *TP53* mutations are likely to be more frequent in BCBM compared to BC (59% vs. 39%) [22].

Molecules and molecular mechanisms that control and regulate critical steps of the brain metastatic process are complex and subject of several studies. In vitro assays demonstrated that ERBB2-ERBB3 dimers promote BBB transendothelial migration fostered by a chemotaxic signal of their ligand NRG1 [23]. In brain and lung metastatic BC, COX2, EGFR, and HBEGF have been identified as promoting factors of extravasation through nonfenestrated blood vessels and of subsequent colonization [24]. An in vivo study demonstrated that brain metastatic cancer cells interact with the brain microenvironment to promote metastases [25]. Upregulated genes that support establishment of brain metastases include *CXCR4*, *PLLP*, *TNFSF4*, *VCAM1*, *SLC8A2*, and *SLC7A11*. Co-culture experiments demonstrated that PCDH7 directly interacts with GJA1, both which are known to be expressed in TNBC with brain metastatic behavior, to assemble functional gap junctions between cancer cells and astrocytes resulting in promoting brain metastasis via a paracrine activation loop [26]. A neuronal lineage cell reprogramming expression signature including upregulation of *SNAP25*, *SNAP91*, and *BSN* has been detected in xenograph brain tumors originating from human cell lines including a breast cell line with a preference to metastasize to the brain [27].

In the present study, we used complementary techniques to comprehensively analyze the tumor genetics of three BCBM with a focus to identify new molecular biomarkers and targets. We utilized for expression analysis whole transcript arrays that cover on average each exon of a gene with a probe. A number of studies have investigated expression profiles related to BCBM or to different steps of the brain metastatic process using various kinds of samples/model systems, methodologies/techniques, and comparison groups rendering it difficult to identify common gene expression signatures [22, 24, 25, 28–34].

## Methods

### Tumor samples

Tumor samples from three consecutive BCBM, Jed81_MT, Jed82_MT, and Jed89_MT, were derived from patients who were treated surgically in 2015 at the King Abdulaziz University Hospital, Jeddah. Histopathological diagnosis was performed on established criteria. Age of patients at time of BCBM surgery was 60 years for Jed81_MT, 32 years for Jed82_MT, and 56 years for Jed89_MT, respectively. Time period between primary BC and BCBM was 13 years for Jed81_MT, 10 months for Jed82_MT, and 2 years for Jed89_MT. Other sites of distant metastases were reported for Jed81_MT. The three BCBM were classified as grade III tumors according to the Nottingham grading system. The generated array data set of the 35 samples from the core analysis has been deposited at the Gene Expression Omnibus (GEO) under Accession Number GSE100534 including basic demographic and histopathological data of each case. This GEO submission comprises samples of BC and primary brain tumors (prBT) which were previously included in GEO submissions GSE36295, GSE66463, and/or GSE77259.

### Sample selection for array expression study

Sample selection criterion was to identify probe sets that are significant to the brain metastatic process. The selection procedure was carried out by analysis of variance (ANOVA) using a *p* < 0.05 and fold change (FC) > 2 as described earlier [35] in order to select those samples which have the comparably lowest numbers of significantly differentially expressed probe sets with the three BCBM. Therefore, in the core analysis we established the expression profiles of the three BCBM in relation to the expression profiles of 16 non-brain metastatic BC and 16 prBT. The 16 BC samples were selected from 45 cases of a previous BC study [36]. No histopathologically confirmed brain metastases were recorded for the 16 BC in the available reports. Of the 16 BC, two were grade I, four were grade II, one was grade II/III, six were grade III, and three were ungraded. Routine immunohistochemistry (IHC) staining for ERBB2 revealed score 0 in three, 1+ in five, 2+ in one, and 3+ in three BC. For four BC no IHC scores for ERRB2 were available. The 16 prBT all of meningioma histology, were selected from 56 brain tumor samples of different histological types and for which array expression data were available at our repository. Of the 16 prBT, 12 were WHO grade I, three were grade II, and one was grade III. One grade II prBT was brain invasive and another grade II prBT was a recurrence.

### RNA and array expression processing

Native tumor specimens for array expression analysis were transiently stored in RNALater (Qiagen, Hilden, Germany). Isolation of total RNA and array sample processing were performed as described earlier [37, 38]. In brief, the Agilent 2100 Bioanalyzer (Agilent Technologies, Palo Alto, CA) was employed to assess RNA integrity and integrity number was > 5 in the samples used for differential expression analysis. RNA concentration was determined by using the NanoDrop ND-1000 spectrophotometer (NanoDrop Technologies, Wilmington, DE). All RNA samples were processed using the Ambion WT Expression Kit (Life Technologies, Austin, TX), the GeneChip WT Terminal Labeling and Controls Kit (Affymetrix, Santa Clara, CA), and the Affymetrix GeneChip Hybridization, Wash and Stain Kit. Samples were hybridized for 17 h to Affymetrix Human Gene 1.0 ST GeneChip arrays which interrogate with 764,885 probes 36.079 annotated reference sequences (NCBI build 36). On average, each exon of a gene is interrogated with one probe enabling to analyze expression data on the exon level [39]. The arrays were scanned on a GeneChip Scanner 3000 7G. Probe cell intensity data (CEL files) were generated by the GeneChip Command Console Software (AGCC).

### Array expression analysis

The CEL files were imported to Partek Genomics Suite version 6.6 (Partek Inc., Chesterfield, MO) using default settings. QC metrics tables and QC graphical reports served as quality assessment of array expression experiments. The lists of differentially expressed probe sets were generated by ANOVA using either a *p* < 0.05 and FC > 2 or using, where indicated, the more stringent criterion of the false discovery rate (FDR) *p*-value (step-up method) < 0.05 and FC > 2. Principal component analysis was utilized to illustrate overall variance in gene expression between samples or groups of samples. Average linkage hierarchical clustering was performed by using Spearman’s correlation as a similarity matrix. Venn diagrams were generated to display genes that intersect or non-intersect between groups of differentially expressed probe sets. The gene ontology (GO) enrichment tool was employed in the gene expression workflow to group significantly expressed genes into functional categories. The gene enrichment score utilizes the Fisher`s exact test to determine the level of differential gene expression in a functional category. Alternative splicing analysis was applied to identify samples with differentially expressed exons.

### Functional network and pathway analysis

Biological significance of expression data was interpreted by using the Ingenuity Pathways Analysis software (IPA; build version 338830M) (Ingenuity Systems, Redwood City, CA) that uses the Ingenuity Knowledge Base as a reference data set. Direct and indirect molecular relationships were included in the analysis settings. Significance of relationships between analyzed data set molecules and functional frameworks prebuilt or generated de novo by IPA was indicated by Fisher’s exact test *p*-values. The Molecule Activity Predictor was employed to predict expression effects/coherence of expression effects of a molecule on other pathway or network molecules. The canonical pathway workflow was employed to identify molecules from the uploaded data set that are co-expressed in a directional, up- to downstream, pathway. Network analysis was employed to explore significance of fit between molecules of the uploaded data set and networks related to specific diseases and functions. The percentage and number of uploaded molecules matching to molecules of a canonical pathway are a measure for its significance, expressed as a score. Upstream analysis was employed to explain how differences in target gene expression are effected by upstream regulators. The activation z-score predicts the activation states of regulators. Regulator effects analysis was utilized to explain which regulators target differentially expressed genes from the uploaded data set and which kind of downstream effects, i.e. diseases and/or functions are associated. In how far a generated network is consistent with the knowledge base, i.e. either activated or inactivated, is scaled by a consistency score.

### Array copy number variation (CNV) analysis

Array CNV analysis of the three BC brain metastases was performed in duplicate for each case using the CytoScan Reagent Kit according to the manufacturer’s assay protocol (Affymetrix). In brief, tumor DNA from formalin-fixed and paraffin-embedded (FFPE) material was extracted by using the QIAamp DNA FFPE Tissue Kit (Qiagen, Hilden, Germany). The DNA processing steps included DNA restriction, adaptor ligation, PCR amplification and subsequent purification, quantification, fragmentation, and labeling of the PCR products. The hybridization mixtures containing the processed DNA samples were hybridized for 17 h at 50 °C and 60 rpm to Cytoscan HD arrays. The array type interrogates the genome (build HG19) with 750,000 SNP probes and 1.9 million non-polymorphic probes. Subsequently, the arrays were washed stringently, then scanned on a GeneChip Scanner 3000 7G and the GeneChip Command Console Software (AGCC) was utilized to generate probe cell intensity data. Using default parameters the Chromosome Analysis Suite (ChAS) 3.1 was utilized to analyze genomic aberrations including copy number gains and losses, and mosaicisms. The ChAS software utilized NetAffx Genomic Annotation file NA33.1 (build HG19) which contains updated content from DGV, OMIM, and RefSeq. The reported aberrations are from overlapping regions of duplicate experiments.

### Immunohistochemistry

Antibodies (Dako Denmark A/S, Glostrup, DK and Ventana Medical Systems, Tucson, AZ) employed for IHC of the three BCBM consisted of GFAP clone GF2, ERBB2 clone 5B5, and MKI67 clone MIB-1. Quantitative image analysis for proliferation marker MKI67, which is immunoreactive in the late G1, S, G2, and M phases of the cell cycle, was performed on at least 10 high power field images using the ImmunRatio application [40]. Measure of variability was expressed as standard deviation. Four μm sections of formalin-fixed and paraffin-embedded specimens were processed on an automated immunostainer (BenchMark XT, Ventana Medical Systems) according to the manufacturer’s protocols and utilizing the ultraView Universal DAB Detection Kit for detection.

### Whole exome sequencing (WES)

Exome capturing was performed using the Nextera Rapid Capture Expanded Exome Kit according to the manufacturer’s protocol (Illumina, San Diego, CA). This application covers 62 Mb of coding exons, UTRs, and regions of miRNAs. In brief, 50 ng FFPE DNA template from each of the BCBM were tagmented by using the TDE1 enzyme for DNA cleavage and adaptor ligation. Unique index adapters were added to each of the tagmented DNA samples that were subsequently amplified. The generated DNA libraries were purified and then pooled and hybridized to expanded exome oligos. The DNA library was captured by streptavidin magnetic beads. Hybridization and capturing steps were repeated and the enriched DNA library was subsequently amplified. Size distribution of DNA library fragments was analyzed using a high sensitivity DNA chip on an Agilent 2100 Bioanalyzer. Finally, utilizing the NextSeq 500 High Output v2 kit, a denatured aliquot of the DNA library was sequenced on an Illumina NextSeq 500 platform with paired-end reads of 150 bp according to the manufacturer’s protocol. FASTQ files were generated by the Illumina BaseSpace Hub. The FASTQ files were analysed using the Galaxy usegalaxy.org server [41] employing a number of bioinformatics workflow tools. For text manipulation the concatenate data sets tail-to-head (cat) (Galaxy 0.1.0) and for format conversion the FASTQ Groomer were utilized (Galaxy 1.0.4) [42]. Sequence mapping was performed with BWA for Illumina (Galaxy 1.2.3) using build HG19 canonical as reference genome [43]. FreeBayes bayesian genetic variant detector (Galaxy 1.0.2.29-3) was employed to detect polymorphisms and indels in the target region defined by the corresponding BED file [44]. The generated VCF files were uploaded to the Illumina BaseSpace Hub and variants were annotated and classified according to their biological significance using the VariantStudio App 1.0.0. Major filter settings included read depth > 10, no reported global and population frequencies (de facto = 0), and variant calling of mutations, comprising frameshift, stop gained or lost, initiator codon, inframe insertion or deletions, splice, and missense mutations, the latter if scored towards a deleterious and/or damaging variant by SIFT and Polyphen bioinformatic tools [45]. To minimize possible false positive results, the quality score was set on > 100 excluding indicated mutations with quality scores between 0 and 100. A number of mutations were listed in the Single Nucleotide Polymorphism Database (dbSNP) [46] and/or in the Catalogue Of Somatic Mutations In Cancer (COSMIC) [47].

### Mutational analysis

Mutational analysis was performed on FFPE DNA templates with minor modification according to our standard protocols [48]. PCR and sequencing primers are listed in Table 1. For direct sequencing, the purified PCR products were subjected to cycle sequence reactions using the BigDye Terminator V3.1 Cycle Sequencing kit (Applied Biosystems, Foster City, CA). Purified sequencing products were finally resolved by capillary electrophoresis on an ABI PRISM 3130 Sequencer.

**Table 1:** Primer sequences used for conventional sequencing

Gene	Forward primer sequence (3′–5′)	Reverse primer sequence (3′–5′)	Product size (bp)
*LOXHD1*	AACACCTATGAGGTTCAGG	GTTGGACTTGTCTGACTTC	134
*ERBB4*	AACCTGGAGATAACCAGC	CAAGGCATATCGATCCTC	169
*CASP7*	TCGCTTTGGGCTCTTCCA	TGCAGTTACCGTTCCCAC	213

## Results

### Genes differentially expressed in BCBM vs. BC and prBT

**Fig. 1:**
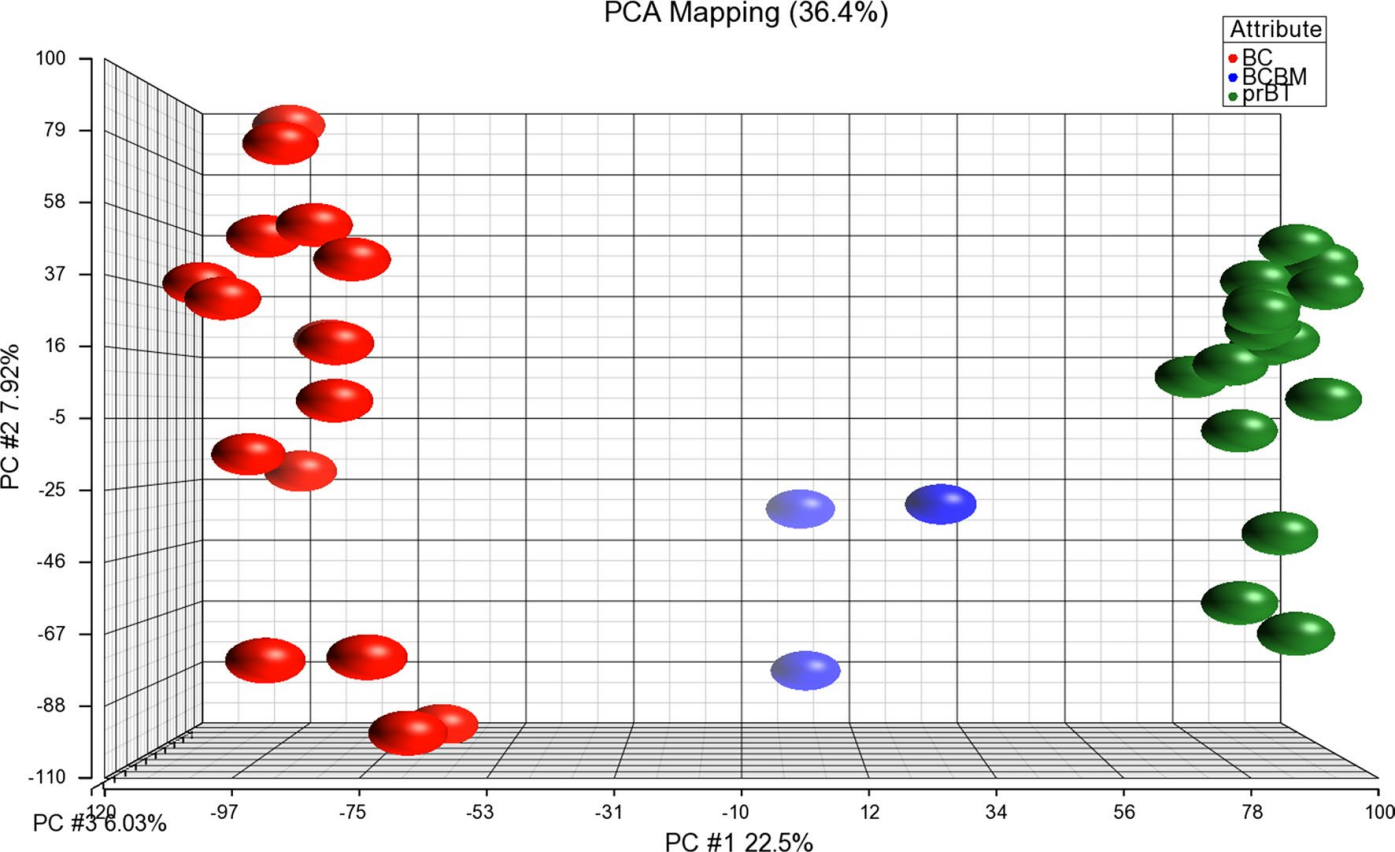
PCA 3D scatter plot as a dimensional, distance-related measure to illustrate similarity of expression profiles of samples, indicated by dots. The three BCBM cluster between the 16 BC and 16 prBT. Sample colors are indicated in the color scheme legend

**Fig. 2:**
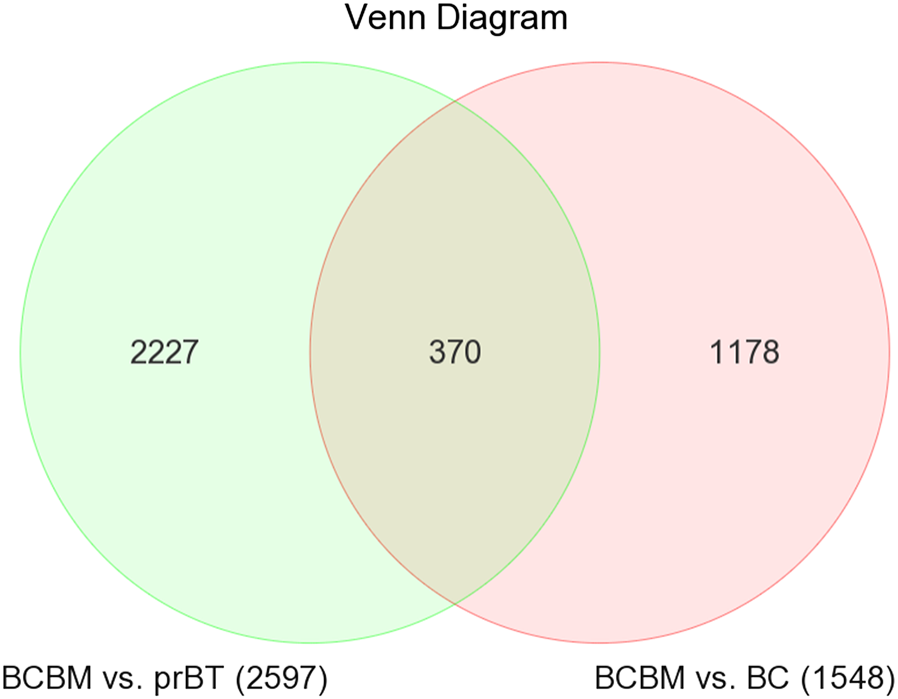
Venn diagram displaying differentially expressed probe sets that intersect or non-intersect between different comparison groups. Applying a FDR *p*-value < 0.05 and FC > 2.0 resulted in 370 differentially expressed probe sets that intersect between the two comparison groups BCBM vs. BC and BCBM vs. prBT (Additional file 1). The 370 probe sets include a number of annotated genes, which are represented by two or more probe sets. Numbers of probe sets that are differentially expressed in each comparison group are given in parentheses

**Fig. 3:**
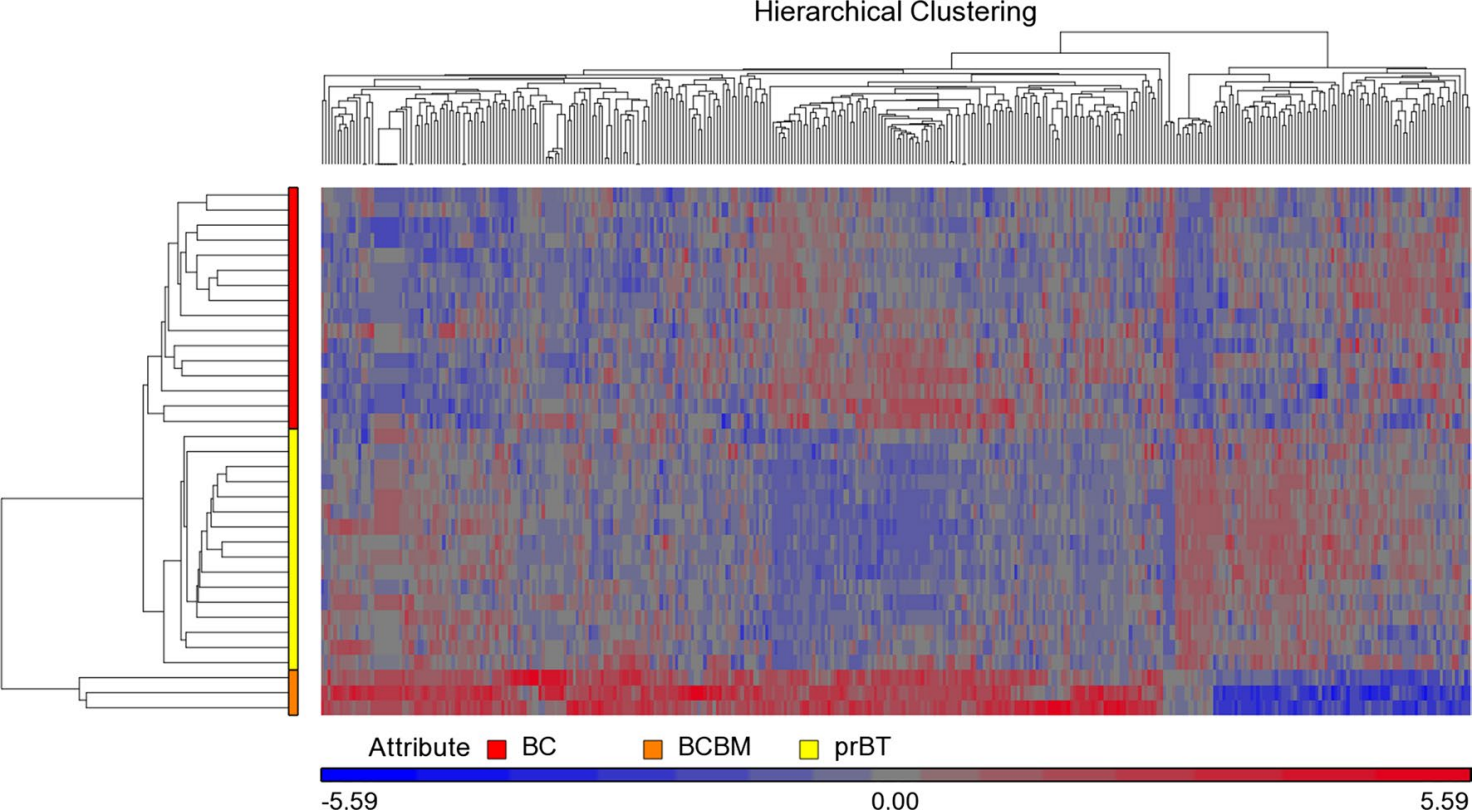
Hierarchical cluster analysis for BCBM, BC, and prBT displaying separate branching of the three sample groups. Analysis is based on 370 differentially expressed probe sets (Additional file 1). Group colors are indicated in the corresponding scheme legend

We performed an expression array study to establish expression profiles of three BCBM that differentiate them from those of 16 selected BC and 16 selected prBT. Similarity of expression profiles of all 35 samples is illustrated by a distance related matrix in a principal component analysis (PCA) 3D scatter plot showing that the three BCBM define a separate cluster between BC and prBT (Fig. 1). From the intersecting area of the two comparison groups BCBM vs. BC and BCBM vs. prBT, a compilation of 370 differentially expressed probe sets (FDR *p* < 0.05 and FC > 2) was established that primarily distinguishes the three BCBM from both, BC and prBT (Fig. 2; Additional file 1). Hierarchical cluster analysis on the 370 probe sets illustrates separate clustering of the three sample groups (Fig. 3). The 370 probe sets were in the majority upregulated in the three BCBM when compared to BC (76% vs. 24%) or compared to prBT (74% vs. 26%). Upregulated genes include, e.g. *BCL3*, *BNIP3*, BNIP3P1, *BRIP1*, *CASP14*, *CCNE2*, *CDC25A*, *CDC45*, *DMBT1*, *E2F1*, *EIF4EBP1*, *HILPDA*, *IDH2*, *MRPL13*, *MT*-*TK*, *MYCN*, *PPP1R14A*, *PPP1R1B*, *RAB3D*, *RAD51*, *RAD54L*, *RASGRF1*, *RRAGD*, and *VDR*. Downregulated genes include, e.g. *ARHGAP24*, *C1S*, *CDON*, *CLK1*, *DRAM1*, *GJA1*, *JAK2*, *KITLG*, *RABGAP1L*, and *RASSF8*. A majority of non-coding RNAs were represented by small nucleolar RNAs (snoRNAs) that included *SNORA1*, *SNORA2A*, *SNORA9*, *SNORA10*, *SNORA22*, *SNORA24*, *SNORA30*, *SNORA37*, *SNORA38*, *SNORA52*, *SNORA71A*, *SNORA71B*, *SNORA71C*, *SNORD13P2*, *SNORD15A*, *SNORD34*, *SNORD35A*, *SNORD41*, *SNORD53*, and *SCARNA22*. Other non-coding RNAs comprised a number of small nuclear RNAs (snRNAs) as, e.g. *RNU4ATAC18P*, *RNU6*-*1199P*, and *RNU6*-*447P*. All snoRNAs and the vast majority of snRNAs were comparably overexpressed in BCBM. The long noncoding RNA *TERC* was same like comparably upregulated in BCBM. Expression of a number of cancer associated genes was studied on the exon level to assess possible exon splicing events that are not detected on the gene level. These genes comprise *BRCA1*, *BRCA2*, *ERBB2*, *TP53*, *ESR1*, *PGR*, *SNORD116*-*4*, *MKI67*, *VDR*, and *BCL3* (Additional file 2A–J).

### Biofunctional prediction analysis

**Fig. 4:**
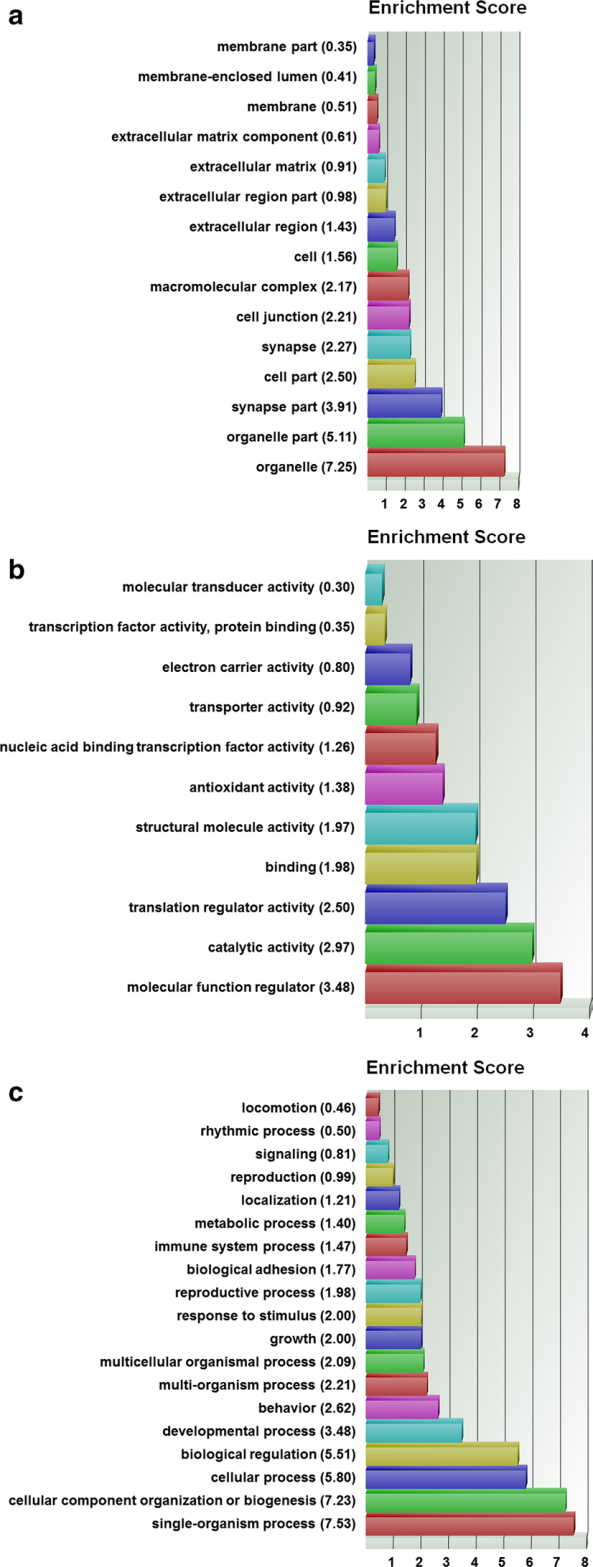
GO enrichment analysis for the 370 differentially expressed probe sets (Additional file 1). The functional categories are ranked according to their *p*-values. **a** The categories organelle, organelle part, and synapse part were most overrepresented in the cellular component domain. **b** The most overrepresented categories in the molecular function domain were molecular function regulator, catalytic activity, and translation regulator activity. **c** The dominant categories in the biological process domain were single-organism process, cellular component organization or biogenesis, and cellular process

**Fig. 5:**
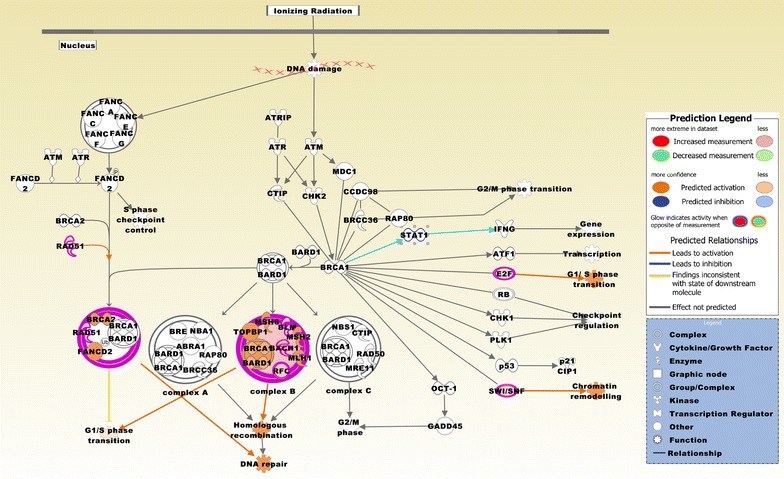
The top canonical pathway is entitled, role of BRCA1 in DNA damage response. The pathway is based on the list of 370 differentially expressed probe sets and is displaying the expression values derived from the BCBM vs. BC comparison group (Additional file 1). Molecules that are comparably upregulated in BCBM include RAD51, DPF1, RFC4, E2F1, BRIP1, and BLM. The pathway was overlaid with the Molecule Activity Predictor to precalculate further molecular effects, as outlined in the prediction legend

**Fig. 6:**
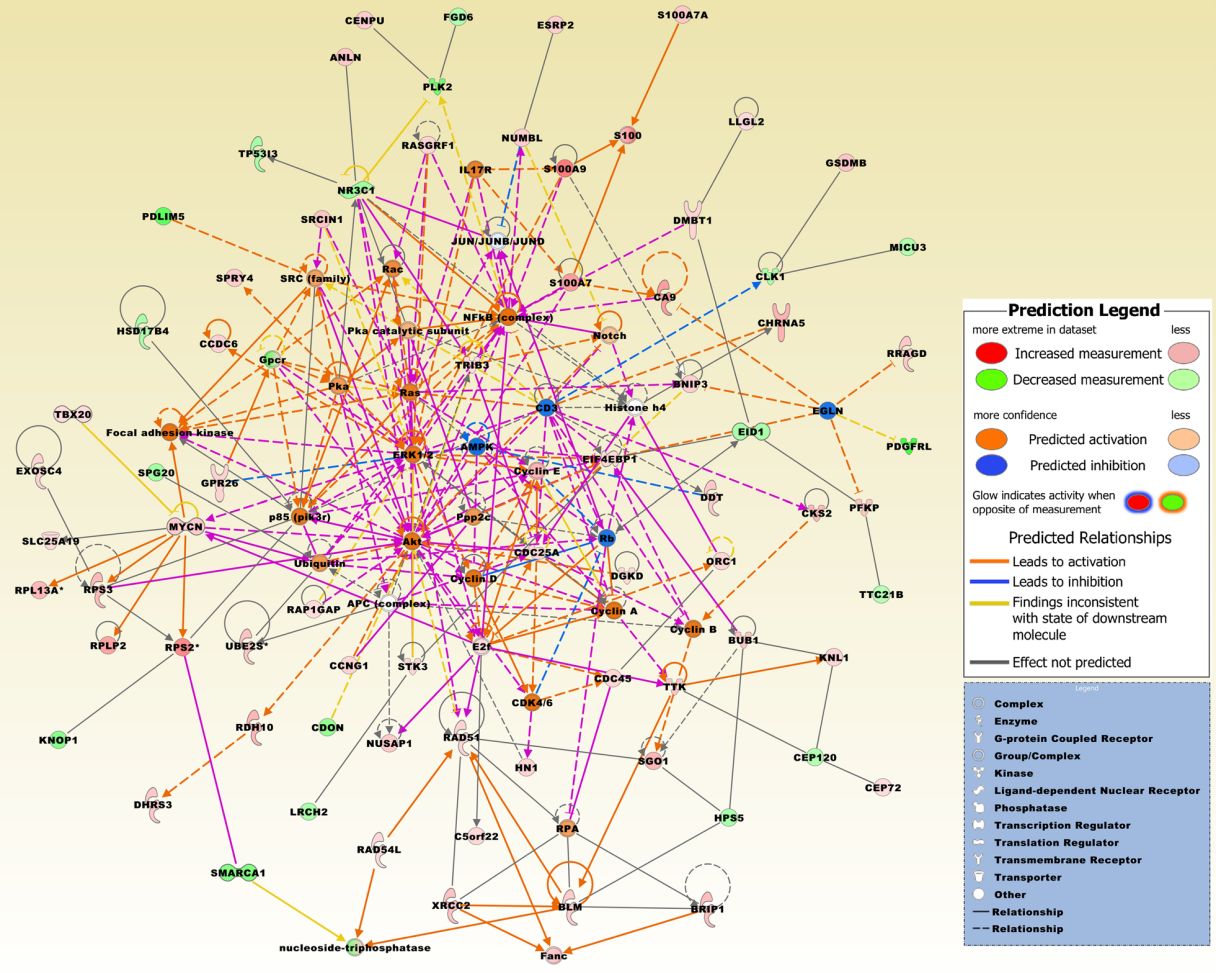
The top three merged networks based on the 370 probe sets comprise molecules related to cell cycle, DNA replication, recombination, and repair, cellular assembly and organization, connective tissue and developmental disorders, gastrointestinal and inflammatory disease and inflammatory response (Table 2). Expression values are derived from the comparison group BCBM vs. BC (Additional file 1). Upregulated molecules include ANLN, BLM, BNIP3, BRIP1, BUB1, C5orf22, CA9, CCDC6, CDC25A, CDC45, CENPU, CEP72, CHRNA5, CKS2, DDT, DGKD, DMBT1, E2f, E2F1, EIF4EBP1 (alias 4EBP1), ESRP2, EXOSC4, GPR26, GSDMB, HN1, LLGL2, KNL1 (CASC5), MYCN, NUSAP1, ORC1, PFKP, RAD51, RAD54L, RAP1GAP, RASGRF1, RDH10, RRAGD, RPL13A (SNORD34, SNORD35A), RPLP2 (SNORA52), RPS2 (SNORA10), RSP3 (SNORD15A), S100A7, S100A7A, S100A9, SGO1 (SGOL1), SLC25A19, SPRY4, SRCIN1, STK3, TBX20, TRIB3, TTK, UBE2S, and XRCC2. Downregulated molecules include CCNG1, CDON, CEP120, CLK1, DHRS3, EID1, FGD6, HPS5, HSD17B4, KNOP1, LRCH2, MICU3, NR3C1, NUMBL, PDGFRL, PDLIM5, PLK2, SMARCA1, SPG20, TP53I3, and TTC21B. Integrative network molecules comprise Akt, AMPK, APC (complex), CD3, CDK4/6, Cyclin A, Cyclin B, Cyclin D, Cyclin E, E2f, EGLN, ERK1/2, Fanc, Focal adhesion kinase (FAC), Gpcr, Histone h4, IL17R, JUN/JUNB/JUND, NFkB (complex), Notch, nucleoside-triphosphatase, Ppp2c, Rb, RPA, S100, and Ubiquitin. Of notice, key nodes as Akt, ERK1/2, NFkB, and Ras were in a predicted activation stage. Asterisks mark molecules with more than one probe set. The pathway was overlaid with the Molecule Activity Predictor to precalculate further molecular effects, as outlined in the prediction legend

**Fig. 7:**
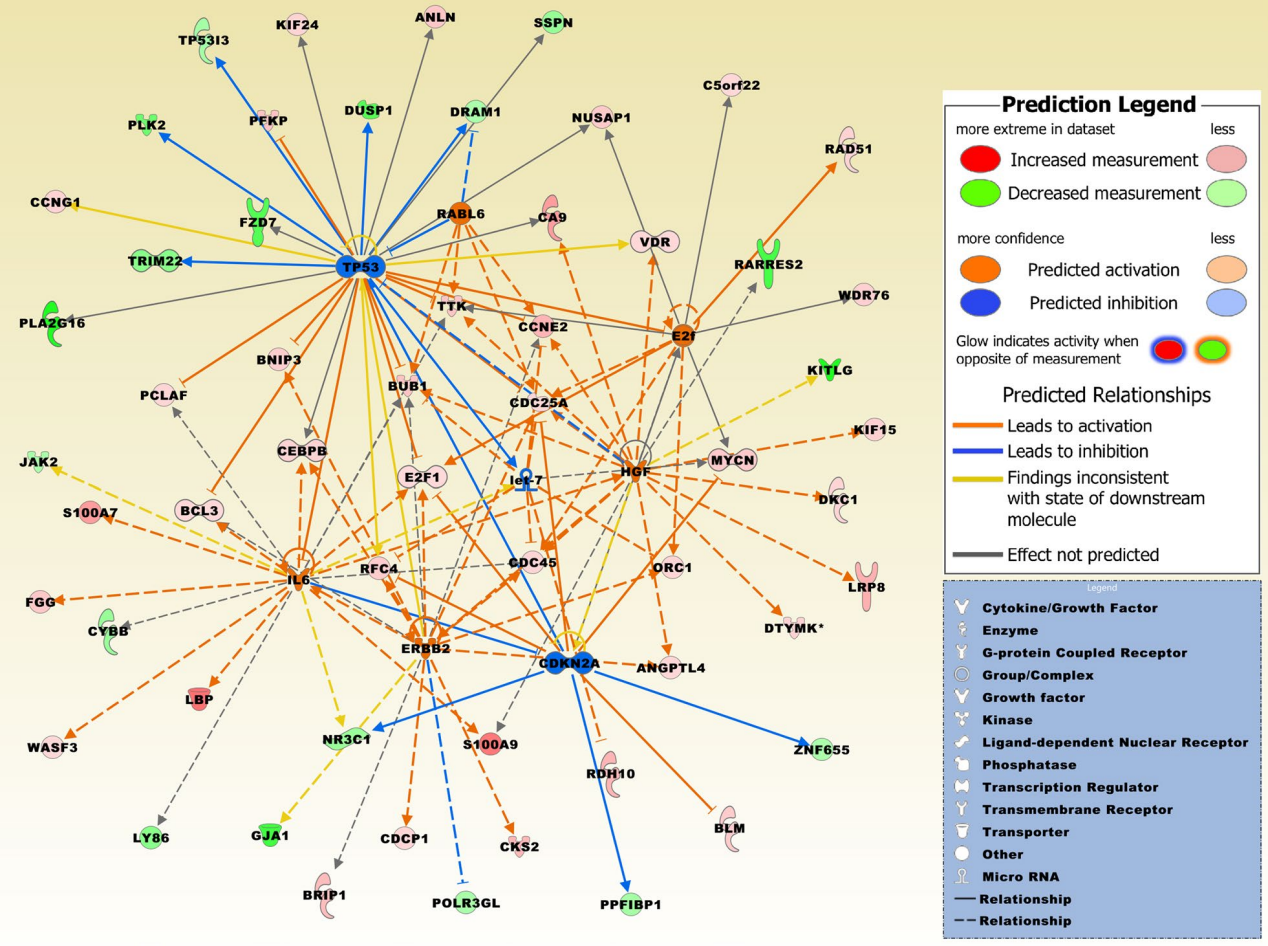
A merged network based on eight upstream regulators, of which E2f, ERBB2, IL6, HGF, and RABL6 were in a predicted activation state (z-score > 2) and let-7, TP53, and CDKN2A were in a predicted inhibition state (z-score < - 2). P-value of overlap for all upstream regulators is < 0.0029. The network is based on the list of 370 differentially expressed probe sets and displays the expression values derived from the comparison group BCBM vs. prBT (Additional file 1). Upregulated molecules include ANGPTL4, ANLN, BCL3, BLM, BNIP3, BRIP1, BUB1, C5orf22, CA9, CCNE2, CCNG1, CDC25A, CDC45, CDCP1, CEBPB, CKS2, DKC1, DTYMK, E2F1, FGG, KIF15, KIF24, LBP, LRP8, MYCN, NUSAP1, ORC1, PCLAF, PFKP, RAD51, RDH10, RFC4, S100A7, S100A9, TTK, VDR, WASF3, and WDR76. Downregulated molecules include CYBB, DRAM1, DUSP1, FZD7, GJA1, JAK2, KITLG, LY86, NR3C1, PLAZG16, PLK2, POLR3GL, PPF1BP1, RARRES2, SSPN, TRIM22, and ZNF655. Asterisk marks molecule with more than one probe set. The pathway was overlaid with the Molecule Activity Predictor to precalculate further molecular effects, as outlined in the prediction legend

**Fig. 8:**
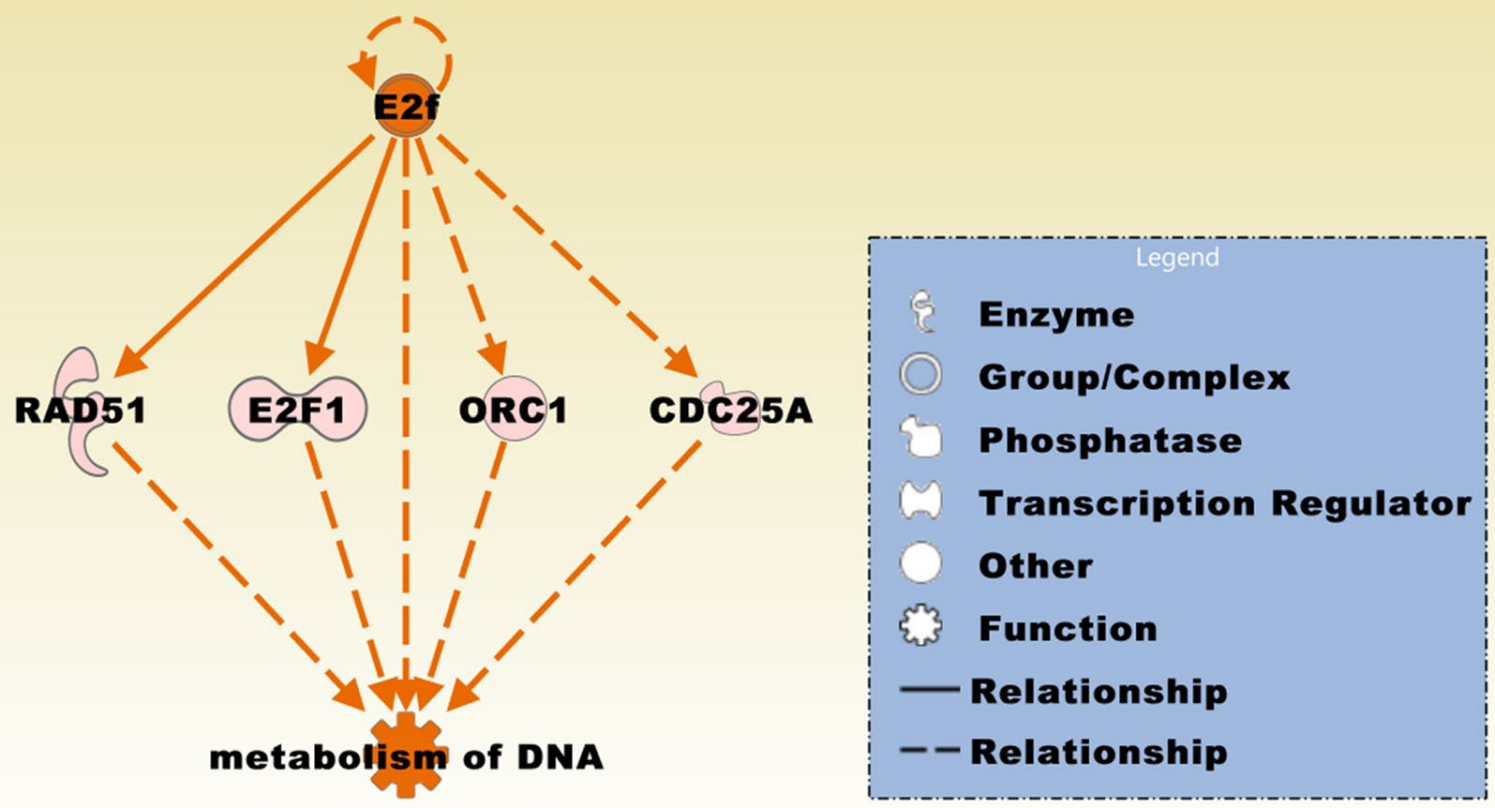
A predicted regulator effects network comprising E2f that regulates RAD51, E2F1, ORC1, and CDC25A from the 370 probe sets and effects metabolism of DNA. Regulator E2f contains the group of E2f transcription factors. The network is in an activation state and has a consistency score 2

Gene ontology (GO) enrichment analysis on the 370 probe sets identified functional groups that were significantly overrepresented in different GO categories (Fig. 4). In the cellular component domain, the categories organelle, organelle part, and synapse part were prevalent. In the molecular function domain, the most significantly related categories were molecular function regulator, catalytic activity, and translation regulator activity. In the biological process domain, the prevalent categories were single-organism process, cellular component organization or biogenesis, and cellular process. Two of the top canonical pathways were entitled, Role of BRCA1 in DNA damage response (Fig. 5) and estrogen-mediated S-phase entry. Other top pathways were involved in biosynthesis of inositol phosphates and degradation of phosphoinositide (Table 2). The top three networks based on the 370 probe sets and merged in Fig. 6 were related to cell cycle, DNA replication, recombination, and repair, cellular assembly and organization, connective tissue and developmental disorders, gastrointestinal and inflammatory diseases, and inflammatory response (Table 2). A number of integrative key nodes including Akt, ERK1/2, NFkB, and Ras were in a predicted activation stage. An upstream regulator network was compiled including the predicted activated upstream regulators E2f, ERBB2, IL6, HGF, and RABL6, and the predicted inhibited upstream regulators let-7, TP53, and CDKN2A (Fig. 7). E2f was the principal regulator effector on the four upregulated molecules RAD51, E2F1, ORC1, and CDC25A causing that the associated function entitled, metabolisms of DNA, was upregulated (Fig. 8).

**Table 2:** Top pathways and networks based on the differentially expressed probe sets

Category	BCBM vs. BC intersecting with BCBM vs. prBT			BCBM shared with prBT		
	*p*-value	Overlap (%)	Score	*p*-value	Overlap (%)	Score
*Top canonical pathways*						
Role of BRCA1 in DNA damage response	1.04E−03	7.7				
d-Myo-inositol (1,4,5,6)-tetrakisphosphate biosynthesis	4.12E−03	5.1				
d-Myo-inositol (3,4,5,6)-tetrakisphosphate biosynthesis	4.12E−03	5.1				
Estrogen-mediated S-phase entry	5.13E−03	12.5				
3-phosphoinositide degradation	6.98E−03	4.7				
EIF2 signaling				3.20E−04	5.7	
Systemic lupus erythematosus signaling				6.10E−03	4.8	
Oxidative phosphorylation				7.43E−03	6.1	
Superoxide radicals degradation				7.94E−03	25.0	
TNFR2 signaling				1.25E−02	10.7	
*Top networks related to diseases and functions*						
Cell cycle, DNA replication, recombination, and repair, cellular assembly and organization			44			
Connective tissue disorders, developmental disorder, gastrointestinal disease			39			
Cellular assembly and organization, inflammatory disease, inflammatory response			37			
Cancer, cell death and survival, organismal injury and abnormalities						51
Hereditary disorder, neurological disease, organismal injury and abnormalities						48
Carbohydrate metabolism, drug metabolism, molecular transport						41

### Genes and biofunctions shared between BCBM and prBT

**Fig. 9:**
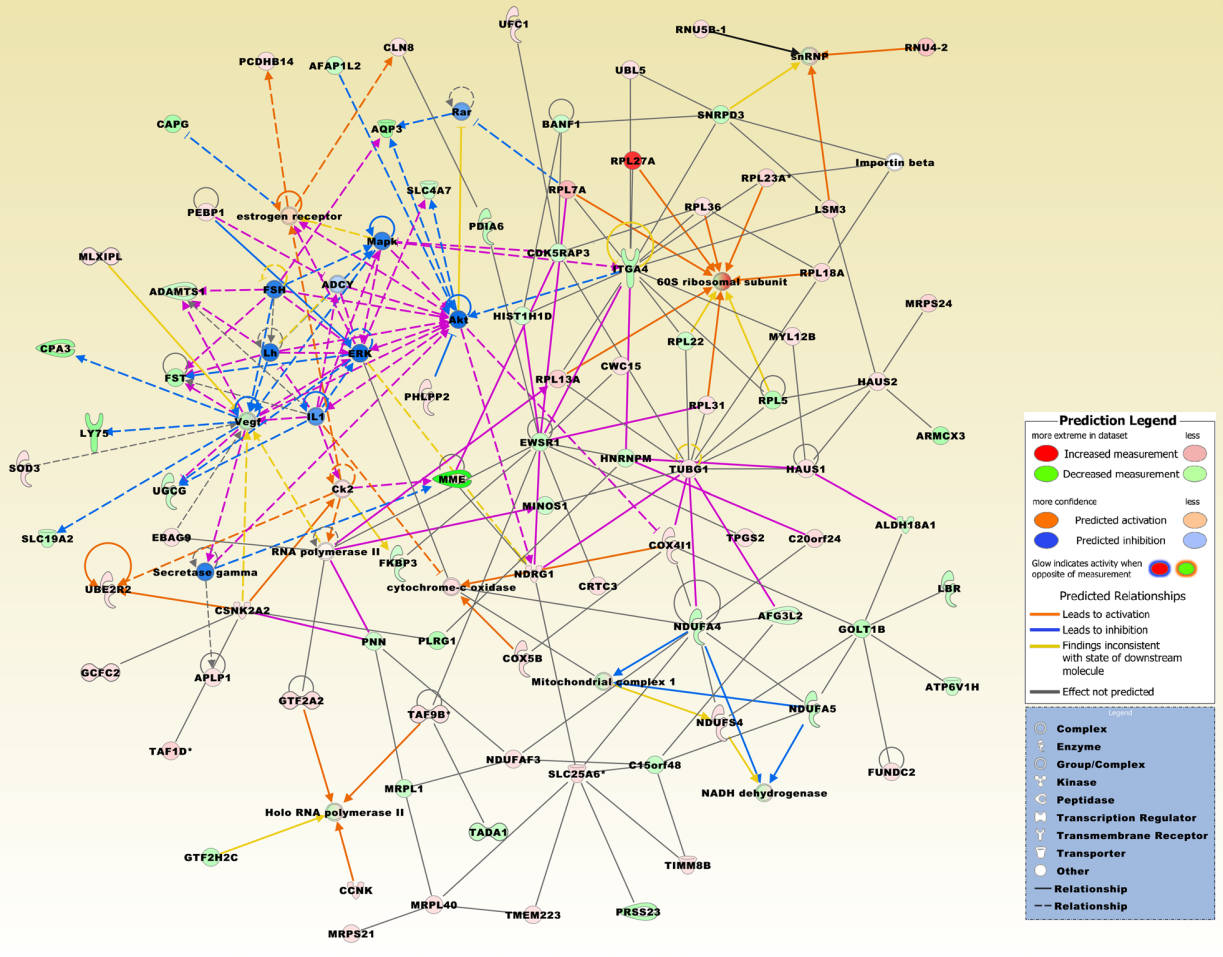
The top three merged networks based on the 643 probe sets that were common between BCBM and prBT but significantly differentially expressed between BCBM and BC include molecules that are related to cancer, cell death and survival, organismal injury and abnormalities, hereditary disorder, neurological disease, carbohydrate and drug metabolism, and molecular transport. Molecules that are upregulated in BCBM compared to BC include, APLP1, C20orf24, CCNK, CLN8, COX4I1, COX5B, CRTC3, CSNK2A2, CWC15, EBAG9, FUNDC2, GCFC2, GTF2A2, HAUS1, HAUS2, LSM3, MLXIPL, MRPL40, MRPS21, MYL12B, NDRG1, NDUFAF3, NDUFS4, MRPS24, PCDHB14, PEBP1, PHLPP2, RNU4-2, RNU5B-1, RPL7A (SNORD36B), RPL13A (SNORD32A), RPL18A (SNORA68), RPL23A (SNORD42A), RPL27A (SNORA45A), RPL31, RPL36, SOD3, TAF1D, TAF9B, TIMM8B, TMEM223, TPGS2, TUBG1, UBE2R2, UBL5, UFC1. Downregulated molecules include ADAMTS1, AFAP1L2, AFG3L2, ALDH18A1, ARMCX3, AQP3, ATP6V1H, C15orf48, CAPG, CDK5RAP3, CPA3, EWSR1, FKBP3, FST, GOLT1B, GTF2H2C (paralogue GTF2H2C_2 in data set), HIST1H1D, HNRNPM, ITGA4, LBR, LY75 (LY75-CD302 readthrough in data set), MINOS1, MME, MRPL1, NDUFA4, NDUFA5, PDIA6, PLRG1, PNN, PRSS23, RPL5 (SNORD21), RPL22, SLC19A2, SLC25A6, SLC4A7, SNRPD3, TADA1, and UGCG. Integrative molecules comprise 60S ribosomal subunit, ADCY, Akt, Ck2, cytochrome-c oxidase, ERK, estrogen receptor, FSH, IL1, Importin beta, Lh, Mapk, mitochondrial complex 1, NADH dehydrogenase, Rar, Holo RNA polymerase II, RNA polymerase II, secretase gamma, snRNP, and Vegf. Asterisks mark molecules with more than one probe set. The pathway was overlaid with the Molecule Activity Predictor to precalculate further molecular effects, as outlined in the prediction legend

**Fig. 10:**
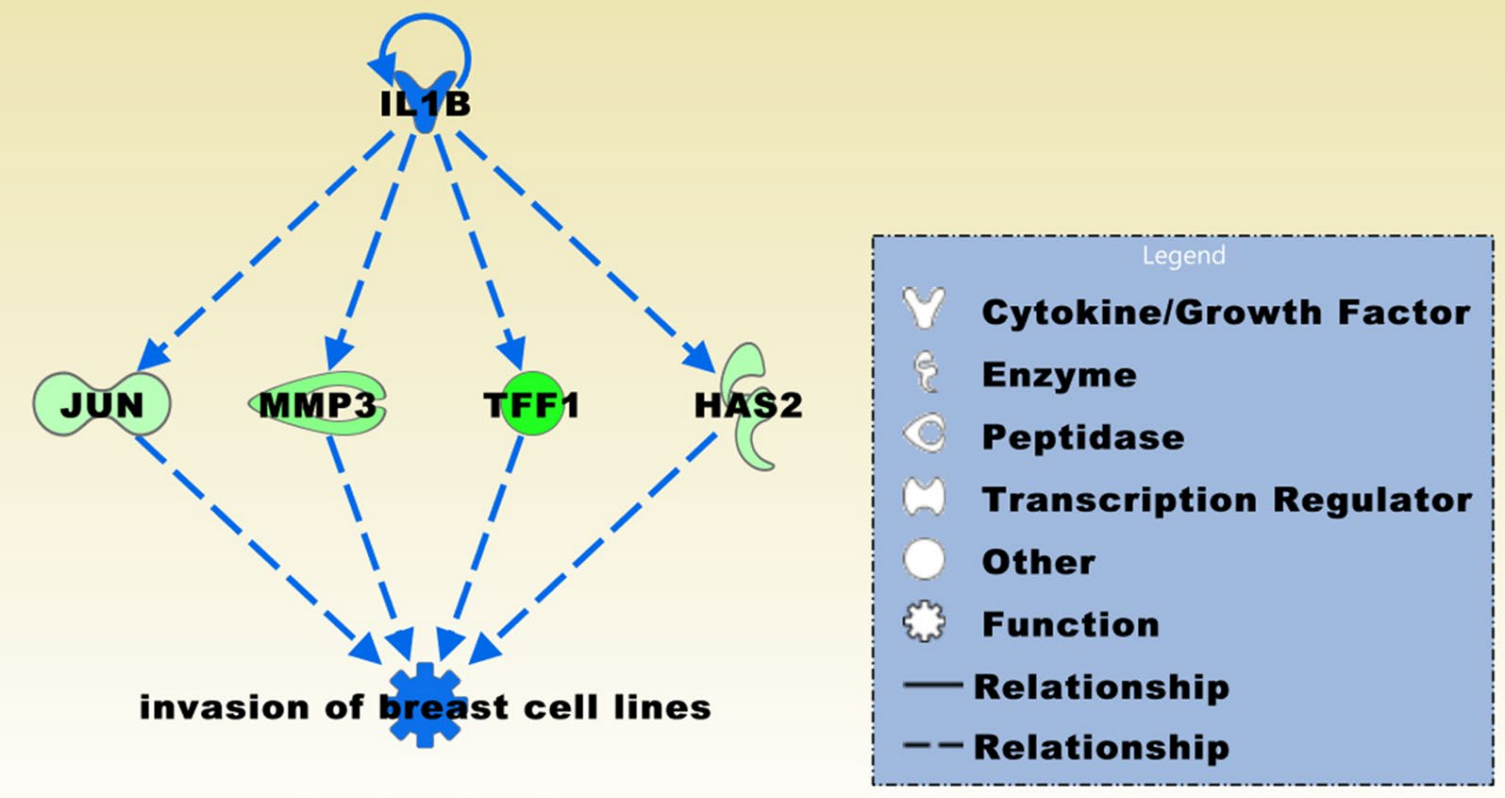
A predicted regulator effects network comprising IL1B that regulates JUN, MMP3, TFF1, and HAS2 from the 643 probe sets and effects invasion of breast cell lines. The network is in an inhibition state and has a consistency score 2

To identify candidate genes that may support the brain metastasis to adapt and expand in the brain microenvironment, we selected in our data compilation for those probe sets that intersect between the comparison groups BCBM vs. BC and BC vs. prBT (FDR *p* < 0.05 and FC > 2.0). In addition, we excluded from the intersecting probe sets those that were included in the list of 585 probe sets derived from the intersection region of the two comparison groups BCBM vs. BC and BCBM vs. prBT (*p* < 0.05 and FC > 2.0). We obtained a list of 643 probe sets that were in the majority upregulated (~ 60%) in BCBM compared to BC. Upregulated genes include, e.g. *ATMIN*, *EIF4A1P2*, *EIF4B*, *CCNK*, *GNG10*, *HIGD2A*, *MALAT1*, *NCS1*, *NDRG1*, *PDPK1*, *RAB3A*, *RAB7A*, *RRAS*, *SOD3*, and *TIMP3*. Downregulated genes include, e.g. *BRMS1*, *CRLF3*, *FOSB*, *GREB1L*, *GPR141*, *JUN*, *MMP14*, *PARP9*, *PDCD4*, *PI4K2B*, *RAB27A*, *SOD1*, and *VEGFC*. In addition, snoRNAs comprised about 7% of the probe sets and the vast majority were comparably upregulated. The top canonical pathway was entitled EIF2 signaling, containing mostly ribosomal proteins, i.e. RPL5, RPL7A, RPL13A, RPL36, RPL22, RPL31, RPL18A, RRAS, RPS14, RPL27A, ATF3, and RPL23A (Table 2). The top three networks based on the 643 probe sets that were common between BCBM and prBT and merged in Fig. 9 were related to cancer, cell death and survival, organismal injury and abnormalities, hereditary disorder, neurological disease, carbohydrate and drug metabolism, and molecular transport (Table 2). Il1B was the top regulator effector on the four downregulated, invasion related molecules JUN, MMP3, TFF1, and HAS2 (Fig. 10). Consequently, the associated function entitled, invasion of breast cell lines was downregulated implying that the invasive behavior of BCBM has been reprogrammed when compared to BC.

### Array CNV analysis

Array CNV analysis was performed in duplicate for each sample revealing highly aberrant genomic profiles of all three BCBM. Important cancer genes affected by CNVs include *TP53*, *BRCA1*, *BRCA2*, *ERBB2*, *IDH1*, and *IDH2* (Table 3). Mosaic-loss of the chromosomal region that comprises *TP53* was found in all three BCBM and may indicate a late cytogenetic event that is only present in subclones. Either *BRCA1* or *BRCA2* were affected in all three BCBM by gain, loss, or mosaic-loss. Amplification of *ERBB2* was detected in Jed82_MT, sustaining IHC results. Mosaic-loss was revealed for the region comprising *IDH1* in Jed81_MT and Jed82_MT whereas gain of *IDH2* was found in Jed89_MT. Conventional sequencing of *IDH1* and *IDH2* hotspot regions indicated no mutation (data not shown).

**Table 3:** CNVs in *TP53*, *BRCA1*, *BRCA2*, *ERBB2*, *IDH1*, and *IDH2*

Case	Copy number state	No. of OMIM genes^1^	Important cancer genes	Array nomenclature^2^
Jed81_MT	Mosaic-loss	226	*TP53*	arr[hg19] 17p13.3p11.2(525-20,011,122)x1-2
Jed82_MT	Mosaic-loss	291	*TP53*	arr[hg19] 17p13.3q12(525-32,273,420)x1-2
Jed89_MT	Mosaic-loss	289	*TP53*	arr[hg19] 17p13.3q11.2(525-31,245,235)x1-2
Jed82_MT	Loss	6	*BRCA1*	arr[hg19] 17q21.31(41,251,930-41,829,105)x1
Jed81_MT	Gain	5	*BRCA2*	arr[hg19] 13q13.1q13.2(32,960,791-34,576,864)x3
Jed89_MT	Mosaic-loss	103	*BRCA2*	arr[hg19] 13q11q14.2(19,436,286-47,693,486)x1-2
Jed82_MT	Gain	23	*ERBB2*	arr[hg19] 17q12q21.1(37,063,504-38,179,492)x4
Jed81_MT	Mosaic-loss	435	*IDH1*	arr[hg19] 2q14.3q37.3(123,839,696-242,275,944)x1-2
Jed82_MT	Mosaic-loss	75	*IDH1*	arr[hg19] 2q32.3q35(192,641,695-216,741,600)x1-2
Jed89_MT	Gain	11	*IDH2*	arr[hg19] 15q26.1(90,592,464-91,543,761)x4

^1^OMIM, online mendelian inheritance in man; ^2^on average 91% overlap of affected regions between duplicate assays

### Immunohistochemistry

**Fig. 11:**
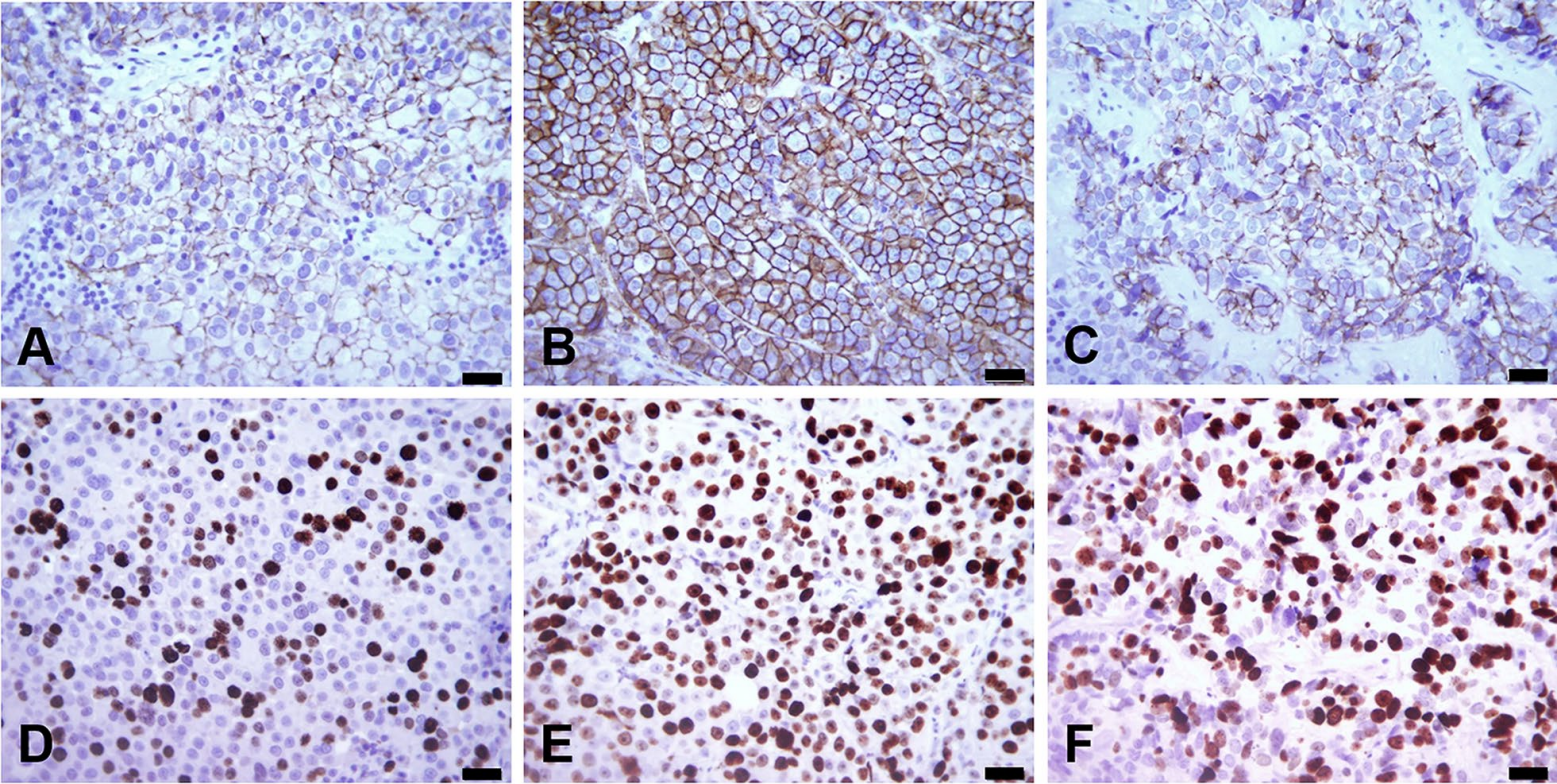
IHC for Jed81_MT (left panel), Jed82_MT (middle panel), and Jed89_MT (right panel). **A**–**C** ERBB2 staining provides score 1+ for complete or nearly complete membrane staining in small focal areas of Jed81_MT and Jed89_MT, and score 3+ for complete intense cell membrane staining in Jed82_MT. **D**–**F** tumor cells stain positive for proliferation marker MKI67 in 21 ± 12.1% of Jed81_MT, in 45 ± 14.7% of Jed82_MT, and in 39 ± 7.8% of Jed89_MT. Black scale bars represent 1 μm. Original magnification, × 400

In all three BCBM, GFAP staining was revealed positive in interspersed astrocytes and in marginal gliosis areas but not in BCBM cells. ERBB2 staining was scored 1+ for Jed81_MT and Jed89_MT and 3+ for Jed82_MT (Fig. 11A–C). Nuclear staining for proliferation marker MKI67 was detected in 21 ± 12.1% of Jed81_MT cells, in 45 ± 14.7% of Jed82_MT cells, and in 39 ± 7.8% of Jed89_MT cells (Fig. 11D–E).

### Whole exome sequencing

**Fig. 12:**
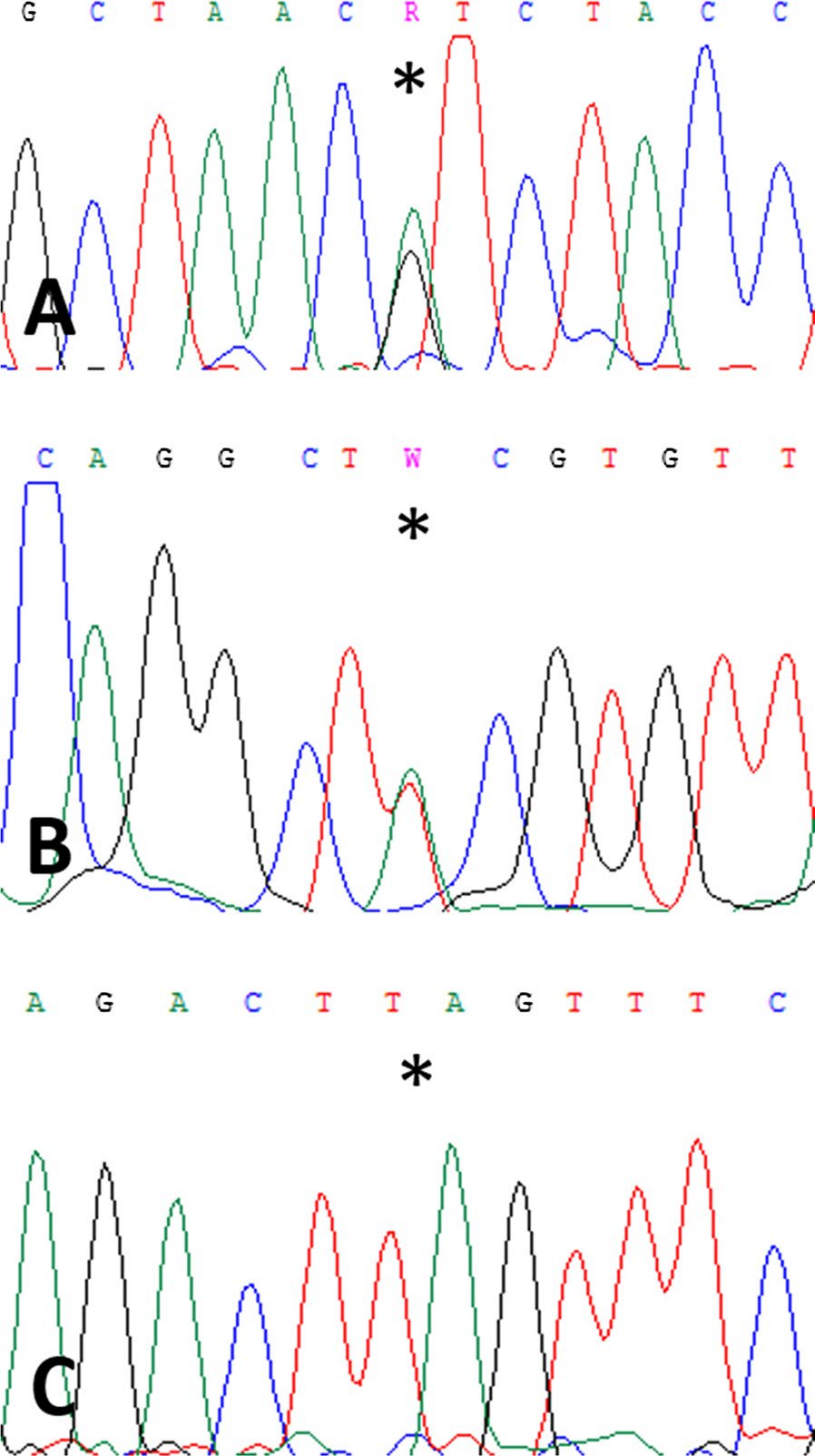
Mutations identified by WES and verified by conventional sequencing. **A** *LOXHD1* missense mutation c.3124G > A; p.Val1042Ile in Jed81_MT. **B** *ERBB4* missense mutation c.257A > T; p.Tyr86Phe in Jed82_MT. **C** *CASP7* frameshift (fs) mutation c.128_130delTTTinsT; p.Leu44SerfsTer70 in Jed89_MT. Asterisks mark missense and frameshift/homozygous deletion mutations, respectively. Sequence reactions from the reverse strand are shown in sense strand view

Based on the chosen parameters, 615 mutations were reported in Jed81_MT, 544 in Jed82_MT, and 461 in Jed89_MT. Read depth for these mutations was on average 50.4 in Jed81_MT, 43.1 in Jed82_MT, and 51.3 in Jed89_MT. Taken together, the three most common mutation types, including those located in splice regions, were missense variants with 61%, followed by frameshift variants with 17%, and stop gained variants with 5%. Mutation types with lower frequencies (1% < range < 5%) included down- and upstream variants, splice region variants (others than listed under the most common types), inframe deletions, and inframe insertions. A comprehensive list of genes affected by mutations includes, e.g. *BCL9L*, *CASP7*, *CDH1*, *ERBB4*, *FLT3*, *FOXD4*, *HEPACAM*, *LOXHD1*, and *PIK3C2G* (Table 4). Conventional sequencing electropherograms are displayed for mutations in *LOXHD1*, *ERBB4*, and *CASP7* (Fig. 12).

**Table 4:** Selection of gene mutations identified by WES

Jed81_MT^1,2^	Jed82_MT^1,2^	Jed89_MT^1,2^	Gene	Read depth	Consequence	HGVSc^3^	HGVSp^4^
he			*ANLN*	11	Inframe insertion	NM_018685.2:c.881_884delCTTCinsCTTCTTC	NM_018685.2:c.881_884delCTTCinsCTTCTTC
he			*BCL9L*	19	Missense	NM_182557.2:c.1276G > C	NP_872363.1:p.Glu426Gln
	he	ho	*CASP7*	18	Frameshift	NM_001267057.1:c.128_130delTTTinsT	NP_001253986.1:p.Leu44SerfsTer70
he			*CDH1*	26	Missense	NM_004360.3:c.1198G > C	NP_004351.1:p.Asp400His
		he	*CDH9*	33	Missense	NM_016279.3:c.643G > T	NP_057363.3:p.Gly215Cys
	he		*CDH10*	128	Missense	NM_006727.3:c.1708G > C	NP_006718.2:p.Asp570His
ho	ho	ho	*CYFIP2*	12, 26, 16	Frameshift	NM_001037333.1:c.280_281delCCinsCCC	NP_001032410.1:p.Gln95ProfsTer15
he			*DAPK1*	19	Missense	NM_004938.2:c.3090G > T	NP_004929.2:p.Gln1030His
	he		*E2F7*	16	Missense	NM_203394.2:c.1292C > T	NP_976328.2:p.Pro431Leu
	he		*ERRB4*	24	Missense	NM_005235.2:c.257A > T	NP_005226.1:p.Tyr86Phe
		he	*ERRB4*	31	Missense	NM_005235.2:c.2621A > T	NP_005226.1:p.Glu874Val
	he		*FLT3*	14	Missense	NM_004119.2:c.2957C > T	NP_004110.2:p.Pro986Leu
		ho	*FOXD4*	50	Frameshift	NM_207305.4:c.1264_1271delGTTTTTTTinsGTTTTTTTT	NP_997188.2:p.Leu424PhefsTer59
		ho	*FOXD4*	13	Frameshift	NM_207305.4:c.755_756delGGinsGGG	NP_997188.2:p.Arg253GlufsTer230
		he	*FOXD4L1*	74	Frameshift	NM_012184.4:c.763_764delGGinsGGG	NP_036316.1:p.Arg256GlufsTer72
		ho	*HEPACAM*	24	Stop gained	NM_152722.4:c.298C > T	NP_689935.2:p.Arg100Ter
	he		*IDH3G*	15	Frameshift	NM_004135.3:c.562_563delAGinsA	NP_004126.1:p.Ser188ThrfsTer2
he			*LOXHD1*	27	Missense	NM_144612.6:c.3124G > A	NP_653213.6:p.Val1042Ile
he			*MAP4K3*	29	Stop gained, splice region	NM_003618.3:c.2539G > T	NP_003609.2:p.Glu847Ter
ho	ho		*MMP12*	27, 19	Frameshift	NM_002426.4:c.630_631delCAinsAAA	NP_002417.2:p.Thr211AsnfsTer261
he		he	*PIK3C2G*	24, 14	Inframe deletion	NM_004570.4:c.384_387delCCCCinsC	NP_004561.3:p.Pro129del
		he	*PPARG*	38	Missense	NM_015869.4:c.1360C > A	NP_056953.2:p.Pro454Thr
		he	*RAD54B*	131	Frameshift	NM_012415.3:c.2732_2733delAGinsA	NP_036547.1:p.Ter911TyrfsTer16
		he	*RASGRP3*	20	Missense	NM_170672.2:c.492G > T	NP_733772.1:p.Glu164Asp

^1^he, heterozygous mutation; ^2^ ho, homozygous mutation; ^3^ HGVSc, Human Genome Variation Society notation in the cDNA; ^4^ HGVSp, Human Genome Variation Society notation in the protein

## Discussion

The tumor biology of BCBM is complex and constitutes a challenge to identify new diagnostic and therapeutic targets that bear the capacity to enhance treatment efficiency of the disease. One of our major findings includes the upregulation of a number of snoRNAs compared to BC and prBT. This discovery is likely to be attributed to the fact that we employed whole transcript arrays on native preserved RNA derived from clinical specimens. Biostatistical tools and complementary molecular genetic techniques as high-density CNV analysis and WES allowed us to identify genes known to be related with BC and/or genes which may gain importance as new molecular biomarkers or targets.

### snoRNAs

snoRNAs represent a conserved class of regulatory RNA molecules that are structurally and functionally categorized mainly into box C/D snoRNAs and box H/ACA snoRNAs. They serve as a scaffold for assemblance of small nucleolar ribonucleoproteins in the nucleolus and are primarily involved in guiding post-transcriptional modification of ribosomal and snRNAs which is necessary for rRNA-controlled protein biogenesis [49–51]. Other functions include regulation of alternative splicing [52]. A number of snoRNAs associate with Cajal-body regions, which assemble to intra- and interchromosomal clusters. The majority of snoRNAs are mapping to introns of protein- and non-protein coding genes. Intergenic human snoRNA promoters are enriched in E-boxes which are binding sites for MYC [53], and in *MYCN* amplified neuroblastomas, expression of several snoRNAs was reported to be higher compared to non-amplified neuroblastomas [54]. *MYCN* was in our BCBM significantly higher expressed than in BC and prBT. An in vitro and in vivo study demonstrated that overexpression of snoRNAs in BC is critical for tumorigenicity and acts as a mechanism of nucleolar modulation of p53 for cancer cell survival [55]. Furthermore, RNA sequencing identified a number of snoRNAs that are associated with overall and/or relapse-free survival in BC [56]. Regulative functions have been described for a number of snoRNAs from our 370 probe sets. For example, among other genes, *SNORD34*, *SNORD35A*, *SNORA38B*, and *SNORA71A* were comparably downregulated in a glioblastoma multiforme (GBM) cell line where expression of the developmental gene *HOXA10* was silenced by siRNA treatment leading to a signal cascade that impaired the homologous recombination pathway and ultimately diminished temozolomide resistance [57]. Furthermore, *SNORA71A* and *SNORA71C* were two of the top upregulated snoRNAs upon retinoic acid and/or thalidomide treatment of GBM [58]. SNORD15A and SNORA38 are known to be involved in regulation of chromatin structure [59]. In MCF7 BC cells, SNORD34 was one of two snoRNAs that was found to bind to the nucleic acid-binding protein YB-1 which itself is associated with unfavorable prognosis of BC patients [60]. Among a number of differentially expressed genes, SNORA2A was identified as a highly expressed transcript in, non-sentinel lymph node positive, sentinel lymph nodes metastasis [61]. These data indicate that the set of snoRNAs which were all upregulated in our BCBM represent new biomarkers with a feasibility to gain diagnostic and therapeutic relevance. Important in the clinical context, as snoRNAs are detectable in blood serum and plasma, quantitative and qualitative detection of snoRNAs in BC patients could become a diagnostic tool to identify patients at risk for brain metastasis [62]. The small Cajal body-specific RNA *SCARNA22* which maps to the intron of the Wolf–Hirschhorn syndrome candidate 1 (WHSC1) gene is known to be involved in cell proliferation and stress response in a subset of multiple myelomas [63]. Besides snoRNAs, a number of snRNAs were comparably upregulated in BCBM which could be attributed to impairment of TP53 mediated repression of snRNA transcription by polymerases II and III [64].

### BC associated genes

BRCA1 exerts critical functions in S-phase activation and DNA damage repair [65]. The BRCA1 canonical pathway genes *BRIP1*, *BLM*, *DPF1*, *E2F1*, *RAD51*, and *RFC4* were in our study comparably higher expressed in BCBM. Recent RNA and protein expression studies revealed a higher RAD51 expression in BCBM compared to matched primary BC [66, 67]. Furthermore, in transplantation mouse models, RAD51 competent primary tumors metastasized to bone and brain, whereas primary tumors depleted of RAD51 showed an inhibition of metastatic seedings [66]. Overexpression of RAD51 brain metastatic cells may be a response mechanism to genotoxic stress caused by neuro-inflammatory microenvironment of the brain [67]. In an experimental model system, cancer stem cells in *BRCA1* mutant TNBC which were resistant to PARP inhibition showed higher RAD51 expression [68]. *RAD51* downregulation by shRNA resulted in growth inhibition of TNBC leading to the suggestion that *RAD51* silencing may increase the efficiency of PARP inhibitors. Higher expression of the BRCA interacting protein BRIP1 was detected in ER and PR negative, ERBB2 positive BC and was associated with unfavourable prognosis [69]. A bioinformatics analysis revealed that DPF1, which is a member of the neuron-specific chromatin remodeling complex, is among the most consistently overexpressed genes in various cancers analysed by the TCGA consortium [70]. In MCF7 BC cells, E2F1 downregulation by the sesquiterpene lactone artemisinin resulted in cell cycle arrest whereas constitutive E2F1 expression reversed the antiproliferative effect showing the critical function of E2F1 in promoting BC growth [71]. *RFC4* and *BLM* are known to be upregulated in *BRCA1* mutant BC [72]. A *BRCA1* deficient-like signature has been identified in ERBB2 positive BCBM compared to non-brain metastatic BC [33]. Acquired *ERBB2* alterations leading to elevated ERBB2 expression are known to be frequently associated with BCBM [73]. A MYC driven accumulation of the oncometabolite 2-hydroxyglutarate has been found in BC, primarily in ER  negative and basal-like types [74, 75]. In our 370 probe sets, the mitochondrial *IDH2* and *MYCN* were upregulated in BCBM.

### Whole exome sequencing

In vitro and in vivo experiments demonstrated that mutant BCL9L impairs CASP2 function in aneuploid colorectal cancer cells resulting in tolerating chromosome missegregation, independently of the functional TP53 status [76]. Missense mutations at codon 400 in *CDH1* and at codon 570 in *CDH10* have been previously reported in gastric carcinoma (COSMIC IDs COSM1159626, COSM20771) and urinary tract carcinoma (COSM1311079), respectively  [47]. Women with a constitutional *CDH1* mutation are at elevated risk for breast cancer [77]. In gastric cancer cells, CYFIP2 silencing resulted in enhanced proliferation and colony formation, decreased apoptosis and induced resistance to 5-FU [78]. E2F7 is a repressor of transcription by interacting with co-repressor CtBP2 and transcription factor E2F1 which is an activator of the G1/S phase [79]. DAPK1 is known as a critical regulator of apoptosis and autophagy [80]. The missense mutation in *FLT3* has been earlier detected in a colon carcinoma and an endometrioid carcinoma (COSM946463). FOXD4 has been described as an essential factor for neuronal differentiation [81]. The frameshift mutation in *FOXD4L1* leading to a truncated variant is affecting its C-terminal repressor function [82]. In non- small cell lung carcinoma, knockdown of HEPACAM expression stimulated cell proliferation, migration, and metastasis [83]. The stop gained mutation in *HEPACAM* identified in Jed89_MT has been previously reported in a breast tumor sample (COSM240098). Same like, the detected *LOXHD1* missense mutation in Jed81_MT has been previously detected in an invasive breast carcinoma (COSM1480342). Expression of PIK3C2G is mainly limited to breast, liver, and prostate and its functions remains to be elucidated [84]. Some of the identified mutations, as those that were recurrently observed, may represent rare constitutional variants, yet may contribute to the genetic burden of BCBM.

### Comparison of expression data to BC with BCBM

To assess in how far expression profiles between our 370 probe sets are shared with those from BC with a known BCBM, we substituted in an ANOVA the three BCBM from our two comparison groups, BCBM vs. BC and BCBM vs. prBT, with three TNBC for which a brain metastatic process has been reported (Dr. S. Ambs et al., pers. comm., Feb., 2017). These three samples (GSM927034, GSM927039, and GSM927040) were derived from GEO submission GSE37751 [75]. ANOVA generated 501 probe sets of which 49 intersected with our 370 probe sets. The 49 probe sets comprise, besides a number of snoRNAs and other non-coding RNAs, several annotated coding genes including *ANGPTL4*, *BCL3*, *CETN3*, *EID1*, *GABARAPL1*, *GRHL2*, *HPS5*, *KLHDC2*, *PLA2G16*, *PLK2*, *SERPINB1*, *SESN3*, *TMX4*, TMEM99, *TRIM22*, *UBAP2L*, *UCP2*, and *WWC2*. With the exception of *GRHL2*, which was comparably downregulated in the 49 probe sets but upregulated in the 370 probe sets, all other intersecting genes were either up- or downregulated in the same direction. ANGPTL4 is a TGFβ target gene in cancer cells and in vitro and in vivo experiments demonstrated that it impairs integrity of vascular endothelial cell layers fostering BC cell passage during lung metastatic process [85]. In mice models, knockdown of Bcl3 in ErbB2 positive BC resulted in decreased cell motility and metastatic progression without affecting primary tumor growth; however, resulted in severe reduction of lung metastatic tumors [86]. Functional downregulation of GRHL2 is known to be associated with elevated cell proliferation; yet, its protein expression has been found to be downregulated at the invasion front of primary breast tumors [14]. GABARAPL1 is known to be involved in autophagy and its higher expression was associated with favorable prognosis in BC with lymph node metastases [87]. Based on its cell cycle regulator function PLK2 is known as a tumor suppressor [88]. TMX4 is a thioredoxin-related molecule that is localized to the ER and involved in protein folding. UBAP2L is a critical factor for hematopoietic stem cell activity and exhibits critical functions in glioma cell growth [89, 90]. In BC cell lines, *UCP2* silencing in combination with cytotoxic treatment led to decrease in cell viability and increase in ROS production, apoptosis, and autophagy [91]. Furthermore, higher UCP2 expression in BC patients corresponded with unfavorable prognosis.

## Conclusions

Using complementary state-of-the-art techniques this study provides a comprehensive overview of the complex tumor biology of BCBM reflecting the challenge to identify effective molecular biomarkers with clinical implications, especially in view that targeted therapy options for BCBM are limited. Among new findings with the capacity to gain clinical relevance is the detection of overexpressed snoRNAs, which represented more than 5% of the 370 probe sets that differentiate BCBM from BC and prBT. The specificity of the probe sets was further demonstrated in the biofunctional analysis showing, e.g. that top merged networks have key nodes as Akt, ERK1/2, NFkB, and Ras in the predicted activation stage. Furthermore, downregulation of four BC cell line invasion markers in a data set that was shared between BCBM and prBT implies reprogramming of the invasive behavior of the BCBM. A number of cancer associated genes were involved in CNVs including mosaic-losses and numerous mutations, some of which in known BC associated genes, were detected by WES.

## Additional files

**Additional file 1.** Probe sets differentially expressed in breast cancer brain metastases (BCBM) compared to breast cancer (BC) and primary brain tumors (prBT).

**Additional file 2.** Exon expression levels for a number of cancer associated genes. A, *BRCA1*; B, *BRCA2*; C, *ERBB2*; D, *TP53*; E, ER1 (*ESR1*); F, PR (*PGR*); G, *SNORD116*-*4*; H, *MKI67*; I, *VDR*; and J, *BCL3*. Comparably low expression of a number of exon probes can be presumbably attributed to splicing events of transcripts; asterisks mark examples. *BCL3* is upregulated on the gene expression level (Additional file 1). Notably, 5` located exons of *ERBB2* are lower expressed than 3` located exons in prBT and expression levels of the two probes covering the *SNORD116*-*4* transcript diverge in BCBM and prBT compared to BC. Exon probes are displayed from 5` (left) to 3` (right) of the transcripts. Blue and red colors in heat maps refer to lower and higher expression, respectively.

## Abbreviations

ANOVA: analysis of variance
BBB: blood brain barrier
BC: breast cancer
BCBM: breast cancer brain metastasis
CNV: copy number variation
FC: fold change
FDR: false discovery rate
GO: gene ontology
GBM: glioblastoma multiforme
IHC: immunohistochemistry
PCA: principal component analysis
prBT: primary brain tumors
snoRNA: small nucleolar RNA
snRNAs: small nuclear RNAs
TNBC: triple negative BC
WES: whole exome sequencing
