# Supplementary material for: Comprehensive molecular biomarker identification in breast cancer brain metastases
Source: J Transl Med. 2017 Dec 29;15:269. doi: 10.1186/s12967-017-1370-x (PMC5747948; doi:10.1186/s12967-017-1370-x)
Supplement: Supplementary file 1 — Additional file 1. Probe sets differentially expressed in breast cancer brain metastases (BCBM) compared to breast cancer (BC) and primary brain tumors (prBT). [file 12967_2017_1370_MOESM1_ESM.docx]

| **Additional file 1: Probe sets differentially expressed in breast cancer brain metastases (BCBM) compared to breast cancer (BC) and primary brain tumors (prBT)** | | | | | | | |
| --- | --- | --- | --- | --- | --- | --- | --- |
|  |  |  |  | **BCBM *vs.* BC** | | **BCBM *vs.* prBT** | |
| **Transcript ID** | **Gene symbol** | **Gene name/**  **assignment** | **Chromosomal location** | **FDR**  **p-value** | **Fold change** | **FDR**  ***p*-value** | **Fold change** |
| 8130565 | RNU4ATAC18P | RNA, U4atac small nuclear 18, pseudogene | 6q25.3 | 2.48E-12 | 8.0662 | 0.000191 | 2.23676 |
| 8155083 | CA9 | carbonic anhydrase IX | 9p13.3 | 1.18E-11 | 5.5012 | 3.04E-11 | 5.15858 |
| 8093576 | SCARNA22 | small Cajal body-specific RNA 22 | 4p16.3 | 8.02E-11 | 5.32975 | 1.94E-09 | 4.2915 |
| 8104731 |  |  | --- | 1.02E-09 | 5.31936 | 0.000276 | 2.23168 |
| 8027363 | ZNF726 | zinc finger protein 726 | 19p12 | 1.09E-09 | 3.36706 | 1.80E-10 | 3.71845 |
| 8028286 | SIPA1L3 | signal-induced proliferation-associated 1 like 3 | 19q13.13 | 1.84E-09 | 3.25983 | 3.00E-13 | 5.42895 |
| 8041168 | SNORD53 | small nucleolar RNA, C/D box 53 | 2p23.2 | 2.58E-09 | 5.20268 | 0.000676 | 2.14081 |
| 7930872 | NANOS1 | nanos homolog 1 (Drosophila) | 10q26.11 | 3.52E-09 | 4.19986 | 3.57E-09 | 4.19609 |
| 8135943 | RNA5SP242 | RNA, 5S ribosomal pseudogene 242 | 7q32.1 | 3.77E-09 | 56.4764 | 0.002 | 5.43931 |
| 8085393 | TMEM40 | transmembrane protein 40 | 3p25.2 | 4.11E-09 | 6.0963 | 2.17E-11 | 9.66517 |
| 7989768 | SNORA24 | Small nucleolar RNA SNORA24 ENSG00000206903 | 15q22.31 | 4.48E-09 | 3.32924 | 1.28E-05 | 2.18005 |
| 8039491 | UBE2S | ubiquitin-conjugating enzyme E2S | 19q13.43 | 7.30E-09 | 3.36799 | 1.95E-06 | 2.4741 |
| 8175336 | CT45A5 | cancer/testis antigen family 45 member A5 | Xq26.3 | 7.57E-09 | 5.49494 | 3.77E-08 | 4.83742 |
| 8066256 | SNORA71B | small nucleolar RNA, H/ACA box 71B | 20q11.23 | 8.41E-09 | 6.20867 | 3.25E-06 | 3.77981 |
| 7911349 | RNU6-1199P | RNA, U6 small nuclear 1199, pseudogene | 1p36.33 | 8.91E-09 | 3.89304 | 3.00E-05 | 2.35757 |
| 8154620 | SNORA30 | Small nucleolar RNA SNORA30 | 9p21.3 | 1.01E-08 | 2.71817 | 5.07E-06 | 2.04348 |
| 7937483 | SNORA52 | small nucleolar RNA, H/ACA box 52 | 11p15 | 1.09E-08 | 5.26321 | 9.18E-05 | 2.64716 |
| 8175353 | CT45A5 | cancer/testis antigen family 45 member A5 | Xq26.3 | 1.40E-08 | 6.27881 | 4.03E-08 | 5.72392 |
| 8170060 | CT45A5 | cancer/testis antigen family 45 member A5 | Xq26.3 | 1.54E-08 | 5.24606 | 5.26E-08 | 4.76066 |
| 8023392 | SNORA37 | small nucleolar RNA, H/ACA box 37 | 18q21.2 | 1.70E-08 | 7.54915 | 0.000241 | 3.06212 |
| 8035838 | ZNF724P | zinc finger protein 724, pseudogene | 19p12 | 1.72E-08 | 5.78926 | 5.85E-07 | 4.31735 |
| 8012958 | UBE2S | ubiquitin-conjugating enzyme E2S | 19q13.43 | 2.36E-08 | 3.38539 | 3.66E-06 | 2.52576 |
| 8175344 | CT45A5 | cancer/testis antigen family 45 member A5 | Xq26.3 | 2.50E-08 | 4.54169 | 1.19E-07 | 4.0511 |
| 7906574 | SUMO1P3 | SUMO1 pseudogene 3 (functional) | 1q22 | 3.44E-08 | 3.75503 | 7.46E-05 | 2.30147 |
| 8081241 | CMSS1 | cms1 ribosomal small subunit homolog (yeast) | 3q12.1 | 3.86E-08 | 4.83111 | 0.001677 | 2.12495 |
| 7911331 | RNU6-447P | RNA, U6 small nuclear 447, pseudogene | 11p15.5 | 4.23E-08 | 4.33718 | 0.000108 | 2.47873 |
| 7924463 | RNU6-447P | RNA, U6 small nuclear 447, pseudogene | 11p15.5 | 4.23E-08 | 4.33718 | 0.000108 | 2.47873 |
| 7927089 | RNU6-447P | RNA, U6 small nuclear 447, pseudogene | 11p15.5 | 4.23E-08 | 4.33718 | 0.000108 | 2.47873 |
| 7945347 | RNU6-447P | RNA, U6 small nuclear 447, pseudogene | 11p15.5 | 4.23E-08 | 4.33718 | 0.000108 | 2.47873 |
| 7998115 | RNU6-447P | RNA, U6 small nuclear 447, pseudogene | 11p15.5 | 4.23E-08 | 4.33718 | 0.000108 | 2.47873 |
| 8031997 | RNU6-447P | RNA, U6 small nuclear 447, pseudogene | 11p15.5 | 4.23E-08 | 4.33718 | 0.000108 | 2.47873 |
| 8102530 | RNU6-447P | RNA, U6 small nuclear 447, pseudogene | 11p15.5 | 4.23E-08 | 4.33718 | 0.000108 | 2.47873 |
| 8137668 | RNU6-447P | RNA, U6 small nuclear 447, pseudogene | 11p15.5 | 4.23E-08 | 4.33718 | 0.000108 | 2.47873 |
| 8077728 | EMC3-AS1 | EMC3 antisense RNA 1 | 3p25.3 | 4.31E-08 | 3.07573 | 2.50E-07 | 2.78933 |
| 8170052 | CT45A5 | cancer/testis antigen family 45 member A5 | Xq26.3 | 5.21E-08 | 6.41821 | 2.51E-07 | 5.54673 |
| 7944869 | SPA17 | sperm autoantigenic protein 17 | 11q24.2 | 5.58E-08 | 4.09785 | 0.00115 | 2.0452 |
| 8132998 |  |  | --- | 5.61E-08 | 4.42576 | 0.001809 | 2.05307 |
| 8116247 | ZNF354A | zinc finger protein 354A | 5q35.3 | 5.68E-08 | 2.80767 | 2.64E-06 | 2.30699 |
| 8170068 | CT45A5 | cancer/testis antigen family 45 member A5 | Xq26.3 | 6.82E-08 | 5.31055 | 7.12E-07 | 4.36157 |
| 8151559 | SLC10A5 | solute carrier family 10, member 5 | 8q21.13 | 8.06E-08 | 4.35797 | 0.000374 | 2.33367 |
| 7986092 | FURIN | furin (paired basic amino acid cleaving enzyme) | 15q26.1 | 8.54E-08 | 2.96298 | 2.57E-05 | 2.16943 |
| 8060286 | DTYMK | deoxythymidylate kinase (thymidylate kinase) | 2q37.3 | 8.79E-08 | 2.49499 | 1.02E-05 | 2.00412 |
| 8077262 | DTYMK | deoxythymidylate kinase (thymidylate kinase) | 2q37.3 | 8.79E-08 | 2.49499 | 1.02E-05 | 2.00412 |
| 8028323 | ENSG00000200209 | Y_RNA ENSG00000200209 | 19q13.2 | 1.13E-07 | 3.86189 | 6.67E-07 | 3.4128 |
| 8009832 | TSEN54 | TSEN54 tRNA splicing endonuclease subunit | 17q25.1 | 1.13E-07 | 2.02427 | 9.85E-09 | 2.21663 |
| 8168432 |  |  | --- | 1.28E-07 | 4.69805 | 0.004234 | 2.02698 |
| 8139816 |  |  | --- | 1.49E-07 | 2.8616 | 6.98E-05 | 2.04934 |
| 8168079 | RNA5SP506 |  | Xq13.1 | 1.51E-07 | 12.5541 | 0.000836 | 4.03532 |
| 8180370 |  |  | --- | 1.57E-07 | 2.65347 | 6.92E-07 | 2.46047 |
| 8016578 | SLC35B1 | solute carrier family 35, member B1 | 17q21.33 | 1.66E-07 | 2.80237 | 6.88E-05 | 2.02968 |
| 8073612 | TSPO | translocator protein (18kDa) | 22q13.31 | 1.73E-07 | 3.20033 | 0.000308 | 2.0318 |
| 7948987 | PLA2G16 | phospholipase A2, group XVI | 11q12.3 | 1.84E-07 | -6.07822 | 4.21E-05 | -3.6439 |
| 8092000 | TERC | telomerase RNA component | 3q26 | 2.23E-07 | 7.25606 | 6.73E-06 | 5.0801 |
| 8121130 |  |  | 6q15 | 2.35E-07 | 3.9109 | 0.001594 | 2.05494 |
| 8087780 | RNA5SP132 | RNA, 5S ribosomal pseudogene 132 | 3p21.2 | 2.97E-07 | 3.85067 | 0.000987 | 2.13418 |
| 8113726 | PPIC | peptidylprolyl isomerase C | 5q23.2 | 3.21E-07 | -3.72843 | 0.000205 | -2.35938 |
| 8160771 | KIF24 | kinesin family member 24 | 9p13.3 | 3.37E-07 | 2.43918 | 2.04E-10 | 3.56075 |
| 7919197 | POLR3GL | RNA polymerase III subunit G like | 1q21.1 | 3.43E-07 | -2.26955 | 5.48E-06 | -2.00773 |
| 8108166 | TXNDC15 | thioredoxin domain containing 15 | 5q31.1 | 3.63E-07 | -2.47089 | 1.22E-05 | -2.08112 |
| 8034512 | SNORD41 | small nucleolar RNA, C/D box 41 | 19p13.2 | 4.85E-07 | 11.6829 | 0.006546 | 3.12276 |
| 8140129 | ABHD11 | abhydrolase domain containing 11 | 7q11.23 | 5.82E-07 | 3.54542 | 1.09E-07 | 3.99297 |
| 7935776 |  |  | --- | 6.92E-07 | 6.0124 | 3.35E-08 | 8.20041 |
| 8066260 | SNORA71C | small nucleolar RNA, H/ACA box 71C | 20q11.23 | 7.51E-07 | 3.91606 | 1.30E-09 | 6.51591 |
| 8027247 | ZNF93 | zinc finger protein 93 | 19p12 | 7.64E-07 | 2.40023 | 5.04E-09 | 3.09907 |
| 7905731 | UBAP2L | ubiquitin associated protein 2-like | 1q21.3 | 7.72E-07 | 3.67585 | 8.40E-05 | 2.60614 |
| 8180305 |  |  | --- | 1.13E-06 | 3.492 | 0.001547 | 2.06083 |
| 8096489 | PDLIM5 | PDZ and LIM domain 5 | 4q22 | 1.19E-06 | -4.27011 | 0.002248 | -2.24279 |
| 8007212 | STAT5A | signal transducer and activator of transcription 5A | 17q11. | 1.22E-06 | -4.18315 | 0.001083 | -2.36995 |
| 8152597 | MRPL13 | mitochondrial ribosomal protein L13 | 8q24.12 | 1.26E-06 | 2.88805 | 3.05E-06 | 2.73501 |
| 8066258 | SNORA71A | small nucleolar RNA, H/ACA box 71A | 20q11.23 | 1.29E-06 | 4.61831 | 6.72E-06 | 3.98908 |
| 8170538 | GABRQ | gamma-aminobutyric acid type A receptor theta subunit | Xq28 | 1.54E-06 | 6.76358 | 1.85E-06 | 6.62855 |
| 7916282 | LRP8 | LDL receptor related protein 8 | 1p32.3 | 1.55E-06 | 4.46666 | 6.94E-05 | 3.20084 |
| 7942379 |  |  | --- | 1.58E-06 | 3.60254 | 0.001401 | 2.14604 |
| 8014794 | CACNB1 | calcium channel, voltage-dependent, beta 1 subunit | 17q12 | 1.63E-06 | 4.15106 | 0.000991 | 2.41201 |
| 8009721 | CDR2L | cerebellar degeneration-related protein 2-like | 17q25.1 | 1.67E-06 | 2.66038 | 2.43E-08 | 3.41079 |
| 8028186 | ZNF146 | zinc finger protein 146 | 19q13.1 | 1.77E-06 | 2.26164 | 3.13E-07 | 2.45934 |
| 7998664 | SNORA10 | small nucleolar RNA, H/ACA box 10 | 16p13.3 | 2.22E-06 | 6.71963 | 0.000194 | 4.03136 |
| 8041204 | SNORA10 | small nucleolar RNA, H/ACA box 10 | 16p13.3 | 2.22E-06 | 6.71963 | 0.000194 | 4.03136 |
| 8047069 | INPP1 | inositol polyphosphate-1-phosphatase | 2q32 | 2.29E-06 | -3.42902 | 4.07E-06 | -3.28686 |
| 7938625 | RNA5SP332 | RNA, 5S ribosomal pseudogene 332 | 11p15.2 | 2.61E-06 | 3.59774 | 0.0003 | 2.48734 |
| 7942592 | SNORD15A | small nucleolar RNA, C/D box 15A | 11q13.4 | 2.75E-06 | 3.98727 | 0.000716 | 2.48824 |
| 7986004 | ZNF774 | zinc finger protein 774 | 15q26.1 | 2.91E-06 | 2.39989 | 7.29E-05 | 2.02163 |
| 8139456 | SNORA9 | small nucleolar RNA, H/ACA box 9 | 7p13 | 3.16E-06 | 4.04874 | 3.56E-05 | 3.29308 |
| 8151967 | STK3 | serine/threonine kinase 3 | 8q22.2 | 3.30E-06 | 2.35615 | 5.72E-07 | 2.58398 |
| 8160786 | KIAA1161 | KIAA1161 | 9p13.3 | 3.53E-06 | 2.57915 | 1.13E-09 | 4.19494 |
| 7962827 | SNORA2A | small nucleolar RNA, H/ACA box 2A | 12q13.11 | 3.55E-06 | 4.98926 | 0.000885 | 2.86857 |
| 8103859 | DCTD | dCMP deaminase | 4q35.1 | 3.90E-06 | -2.14531 | 9.56E-06 | -2.05644 |
| 7914807 |  |  | --- | 3.99E-06 | 4.35058 | 0.000131 | 3.16195 |
| 7896752 | MT-TK | mitochondrially encoded tRNA lysine | MT | 4.05E-06 | 7.56297 | 0.007236 | 2.84781 |
| 8165667 | MT-TK | mitochondrially encoded tRNA lysine | MT | 4.05E-06 | 7.56297 | 0.007236 | 2.84781 |
| 8114814 | NR3C1 | nuclear receptor subfamily 3 group C member 1 | 5q31.3 | 4.37E-06 | -2.66205 | 4.68E-08 | -3.52306 |
| 8074168 | LINC01296 | long intergenic non-protein coding RNA 1296 | 14q11.2 | 5.20E-06 | 4.45672 | 1.10E-05 | 4.15453 |
| 8065710 | E2F1 | E2F transcription factor 1 | 20q11.2 | 5.29E-06 | 2.17295 | 2.15E-05 | 2.02881 |
| 7934920 | LIPA | lipase A, lysosomal acid, cholesterol esterase | 10q23.31 | 5.40E-06 | -2.6025 | 3.62E-05 | -2.3202 |
| 8022506 | RNU6-702P | RNA, U6 small nuclear 702, pseudogene | 18q11.2 | 5.95E-06 | 4.33255 | 0.006341 | 2.20579 |
| 8073680 |  |  | 22q13.31 | 6.14E-06 | 19.2141 | 0.003166 | 5.73004 |
| 8108475 | IGIP | IgA-inducing protein | 5q31 | 6.27E-06 | -2.58352 | 2.41E-05 | -2.38168 |
| 7977452 | LINC01296 | long intergenic non-protein coding RNA 1296 | 14q11.2 | 6.38E-06 | 4.70843 | 1.23E-05 | 4.41405 |
| 7901192 | RAD54L | RAD54-like (S. cerevisiae) | 1p32 | 6.56E-06 | 2.12349 | 5.62E-10 | 3.39371 |
| 8134730 | CNPY4 | canopy FGF signaling regulator 4 | 7q22.1 | 6.61E-06 | -2.58837 | 3.15E-05 | -2.35417 |
| 8016128 | GFAP | glial fibrillary acidic protein | 17q21 | 8.72E-06 | 2.72363 | 0.000688 | 2.03951 |
| 7967030 | RNU4-1 | RNA, U4 small nuclear 1 | 12q24.31 | 8.75E-06 | 7.83718 | 0.000206 | 5.11722 |
| 7999903 | KNOP1 | lysine-rich nucleolar protein 1 | 16p12.3 | 8.94E-06 | -3.14454 | 0.000227 | 2.46515 |
| 7989657 | KIAA0101 | KIAA0101 | 15q22.31 | 9.61E-06 | 2.30095 | 3.72E-05 | 2.13691 |
| 8093872 | C4orf6 | chromosome 4 open reading frame 6 | 4p16.2 | 1.13E-05 | 2.11258 | 2.13E-05 | 2.04737 |
| 8014679 | SRCIN1 | SRC kinase signaling inhibitor 1 | 17q12 | 1.13E-05 | 3.28087 | 2.46E-07 | 4.43833 |
| 7973743 | BNIP3P1 | BCL2 interacting protein 3 pseudogene 1 | 14q12 | 1.36E-05 | 2.4167 | 9.41E-06 | 2.47036 |
| 7965721 | RNA5SP366 | RNA, 5S ribosomal pseudogene 366 | 12q23.1 | 1.42E-05 | 3.36267 | 0.00125 | 2.3149 |
| 8038624 | C19orf48 | chromosome 19 open reading frame 48 | 19q13.33 | 1.44E-05 | 2.2787 | 6.65E-07 | 2.70272 |
| 8105495 | PART1 | prostate androgen-regulated transcript 1 (non-protein coding) | 5q12.1 | 1.48E-05 | 2.77246 | 4.78E-08 | 4.12877 |
| 8031931 | RNA5SP473 | RNA, 5S ribosomal pseudogene 473 | 19q13.43 | 1.49E-05 | 6.62607 | 0.004051 | 3.15401 |
| 8151434 | RNU2-54P | RNA, U2 small nuclear 54, pseudogene | 8q21.13 | 1.52E-05 | 2.00086 | 1.91E-06 | 2.20506 |
| 8118207 | SNORA38 | small nucleolar RNA, H/ACA box 38 | 6p21.33 | 1.53E-05 | 3.6525 | 0.0006 | 2.63466 |
| 8064939 | TMX4 | thioredoxin-related transmembrane protein 4 | 20p12 | 1.53E-05 | -3.05792 | 1.15E-05 | -3.12536 |
| 8111629 | NUP155 | nucleoporin 155kDa | 5p13.1 | 1.54E-05 | 2.67844 | 4.21E-06 | 2.9203 |
| 8040419 | MYCN | v-myc avian myelocytomatosis viral oncogene neuroblastoma derived homolog | 2p24.3 | 1.62E-05 | 2.99428 | 5.75E-06 | 3.23319 |
| 8174985 | SMARCA1 | SWI | Xq25q26.1 | 1.62E-05 | -3.35532 | 8.36E-07 | -4.28162 |
| 8027621 | GPI | glucose-6-phosphate isomerase | 19q13.1 | 1.68E-05 | 2.02353 | 1.59E-05 | 2.02896 |
| 8074969 | DDT | D-dopachrome tautomerase | 22q11.23 | 1.73E-05 | 3.13759 | 0.002641 | 2.09296 |
| 8103413 | ENSG00000200521 | Y_RNA ENSG00000200521 | 4q32.1 | 1.74E-05 | 3.46093 | 0.000243 | 2.76233 |
| 7931199 | GPR26 | G protein-coupled receptor 26 | 10q26.13 | 1.74E-05 | 2.55373 | 2.97E-05 | 2.46814 |
| 8005473 | ENSG00000174977 | AC026271.5 ENSG00000174977 | 17p11.2 | 1.81E-05 | 2.42253 | 0.000187 | 2.10151 |
| 8144228 | FLJ36840 | uncharacterized LOC645524 | --- | 1.82E-05 | 3.72161 | 0.000267 | 2.9152 |
| 8081233 | RNU6-1263P | RNA, U6 small nuclear 1263, pseudogene | 3q12.1 | 1.97E-05 | 3.27134 | 0.005737 | 2.01679 |
| 7961524 | ERP27 | endoplasmic reticulum protein 27 | 12p12.3 | 2.00E-05 | 4.24398 | 1.27E-05 | 4.44149 |
| 7933760 | CCDC6 | coiled-coil domain containing 6 | 10q21 | 2.28E-05 | 2.56511 | 0.000675 | 2.04628 |
| 8007108 | TMEM99 | transmembrane protein 99 | 17q21.2 | 2.32E-05 | -3.73037 | 0.000385 | -2.87308 |
| 7965322 | KITLG | KIT ligand | 12q22 | 2.38E-05 | -5.98102 | 0.007241 | -2.82649 |
| 7961900 | ITPR2 | inositol 1,4,5-trisphosphate receptor, type 2 | 12p11 | 2.45E-05 | -3.38621 | 0.000202 | -2.82446 |
| 7983606 | EID1 | EP300 interacting inhibitor of differentiation 1 | 15q21.1 | 2.73E-05 | -2.3162 | 0.000163 | -2.08225 |
| 8083233 | ZIC1 | Zic family member 1 | 3q24 | 2.74E-05 | 6.56154 | 9.51E-05 | -5.55912 |
| 8104220 | CEP72 | centrosomal protein 72kDa | 5p15.33 | 2.81E-05 | 2.00442 | 3.91E-07 | 2.47244 |
| 8113733 | CEP120 | centrosomal protein 120kDa | 5q23.2 | 2.97E-05 | -2.04244 | 6.79E-09 | -3.1444 |
| 8151432 |  |  | --- | 3.01E-05 | 3.88731 | 0.000406 | 3.01458 |
| 7907492 | RABGAP1L | RAB GTPase activating protein 1-like | 1q24 | 3.03E-05 | -2.30479 | 0.000123 | -2.12047 |
| 8036460 | DPF1 | D4, zinc and double PHD fingers family 1 | 19q13.2 | 3.05E-05 | 2.41864 | 9.15E-05 | 2.25687 |
| 8156290 | CKS2 | CDC28 protein kinase regulatory subunit 2 | 9q22 | 3.09E-05 | 4.04449 | 1.30E-07 | 6.97963 |
| 7928516 | SAMD8 | sterile alpha motif domain containing 8 | 10q22.2 | 3.11E-05 | -2.18789 | 3.09E-05 | -2.1889 |
| 8014704 | RN5S440 | RNA, 5S ribosomal pseudogene 440 | 17q12 | 3.16E-05 | 3.301 | 0.004808 | 2.11198 |
| 8150862 | SNORA1 | Small nucleolar RNA SNORA1 | 8q12.1 | 3.50E-05 | 3.09226 | 0.002628 | 2.15224 |
| 8097809 | RN7SKP35 | RNA, 7SK small nuclear pseudogene 35 | 4q31.3 | 3.54E-05 | 2.09791 | 5.03E-05 | 2.059 |
| 8006788 | MLLT6 | MLLT6, PHD finger domain containing | 17p12 | 3.61E-05 | 2.41727 | 0.000201 | 2.16518 |
| 7991374 | IDH2 | IDH2: isocitrate dehydrogenase (NADP(+)) 2, mitochondrial | 15q26.1 | 3.68E-05 | 2.89568 | 4.23E-12 | 10.7639 |
| 7928907 |  |  | --- | 3.73E-05 | 4.00006 | 0.000957 | 2.87054 |
| 7906900 | DDR2 | discoidin domain receptor tyrosine kinase 2 | 1q23.3 | 3.80E-05 | -3.97173 | 2.60E-07 | -6.52729 |
| 7922646 | TOR1AIP2 | torsin A interacting protein 2 | 1q25.2 | 3.93E-05 | -2.86074 | 0.000944 | -2.23343 |
| 7970999 | SPG20 | spastic paraplegia 20 (Troyer syndrome) | 13q13.3 | 4.84E-05 | -2.48614 | 7.73E-09 | -4.50036 |
| 8034304 | ACP5 | acid phosphatase 5, tartrate resistant | 19p13.2 | 5.11E-05 | -3.99105 | 0.005781 | 2.40088 |
| 8145889 | EIF4EBP1 | eukaryotic translation initiation factor 4E binding protein 1 | 8p11.23 | 5.21E-05 | 2.22815 | 6.21E-07 | 2.89517 |
| 8054135 | MGAT4A | mannosyl (alpha-1,3-)-glycoprotein beta-1,4-N-acetylglucosaminyltransferase, isozyme A | 2q11.2 | 5.63E-05 | 3.17116 | 0.0002 | 2.84088 |
| 7949679 | SPTBN2 | spectrin, beta, non-erythrocytic 2 | 11q13 | 5.73E-05 | 2.993 | 4.38E-08 | 5.39447 |
| 8170520 | CNGA2 | cyclic nucleotide gated channel alpha 2 | Xq27 | 5.75E-05 | 2.76655 | 0.000114 | 2.62635 |
| 8098470 | WWC2 | WW and C2 domain containing 2 | 4q35.1 | 6.15E-05 | -2.44735 | 0.000133 | -2.32323 |
| 7912537 | DHRS3 | dehydrogenase/reductase 3 | 1p36.1 | 6.29E-05 | 3.04544 | 0.000591 | -2.51574 |
| 8152828 | GSDMC | gasdermin C | 8q24.21 | 6.37E-05 | 5.33845 | 5.14E-09 | 17.7827 |
| 7901140 | MAST2 | microtubule associated serine/threonine kinase 2 | 1p34.1 | 6.52E-05 | 2.19234 | 3.14E-06 | 2.62081 |
| 8002218 | ESRP2 | epithelial splicing regulatory protein 2 | 16q22.1 | 6.53E-05 | 2.75327 | 1.85E-13 | 14.3741 |
| 7943498 | DYNC2H1 | dynein, cytoplasmic 2, heavy chain 1 | 11q22.3 | 6.83E-05 | -3.2431 | 0.000168 | -2.99096 |
| 8078784 | XYLB | xylulokinase homolog (H. influenzae) | 3p22.2 | 7.15E-05 | 2.37765 | 7.71E-06 | 2.7509 |
| 7905571 | S100A9 | S100 calcium binding protein A9 | 1q21 | 7.25E-05 | 7.58088 | 2.48E-05 | 8.94036 |
| 8164452 | ZDHHC12 | zinc finger, DHHC-type containing 12 | 9q34.11 | 7.93E-05 | 2.03937 | 6.35E-05 | 2.06424 |
| 8148710 | EXOSC4 | exosome component 4 | 8q24.3 | 8.97E-05 | 2.49848 | 0.000456 | 2.22216 |
| 8170992 | DKC1 | dyskeratosis congenita 1, dyskerin | Xq28 | 9.07E-05 | 2.35047 | 2.12E-06 | 3.00968 |
| 8112202 | PLK2 | polo-like kinase 2 | 5q11.2 | 9.08E-05 | -3.90478 | 0.005089 | 2.49852 |
| 8115831 | DUSP1 | dual specificity phosphatase 1 | 5q34 | 9.52E-05 | -4.49252 | 1.03E-05 | -5.82121 |
| 7973002 | LOC101929839 | uncharacterized LOC101929839 | 14q11.2 | 9.60E-05 | 3.72142 | 1.35E-06 | 5.74116 |
| 7990442 | RPP25 | Ribonuclease P/MRP subunit p25 | 15q24.2 | 0.000101 | 2.289 | 4.77E-05 | 2.40246 |
| 7962689 | VDR | vitamin D (1,25- dihydroxyvitamin D3) receptor | 12q13.11 | 0.000102 | 2.18775 | 3.91E-14 | 9.57896 |
| 8086880 | CDC25A | cell division cycle 25A | 3p21 | 0.000115 | 2.33582 | 1.74E-05 | 2.65045 |
| 8036936 | NUMBL | numb homolog (Drosophila)-like | 19q13.2 | 0.000118 | 2.8493 | 0.000792 | -2.42362 |
| 8152606 | SNTB1 | syntrophin beta 1 | 8q24.12 | 0.00012 | 2.29903 | 5.14E-09 | 4.49475 |
| 7979505 | SIX1 | SIX homeobox 1 | 14q23.1 | 0.000122 | 4.89625 | 0.001759 | -3.45724 |
| 8025402 | ANGPTL4 | angiopoietin-like 4 | 19p13.3 | 0.000123 | 2.2623 | 0.000294 | 2.13643 |
| 8059177 | TUBA4A | tubulin, alpha 4a | 2q35 | 0.000133 | 3.03662 | 0.000196 | 2.93231 |
| 8105144 |  |  | --- | 0.000135 | 2.53709 | 0.000152 | 2.51578 |
| 8079462 | NBEAL2 | neurobeachin-like 2 | 3p21.31 | 0.000139 | 2.17337 | 1.02E-08 | 3.94619 |
| 8112388 | TRIM23 | tripartite motif containing 23 | 5q12.3 | 0.00014 | -2.11839 | 0.000162 | -2.09992 |
| 8014812 | STAC2 | SH3 and cysteine rich domain 2 | 17q12 | 0.000143 | 8.37268 | 2.92E-06 | 16.2224 |
| 8063386 | CEBPB | CCAAT/enhancer binding protein beta | 20q13.1 | 0.000145 | 2.49461 | 0.001331 | 2.10772 |
| 8150204 | ENSG00000188512 | RP1-273G13.2 ENSG00000188512 | 8p12 | 0.000147 | 2.28085 | 0.000297 | 2.17487 |
| 8140468 | GSAP | gamma-secretase activating protein | 7q11.23 | 0.000158 | -2.56188 | 5.95E-06 | -3.28606 |
| 8143441 | KIAA1147 | KIAA1147 | 7q34 | 0.00017 | 2.29298 | 0.000601 | 2.10086 |
| 8030364 | SNORD34 | small nucleolar RNA, C/D box 34 | 19q13.3 | 0.000174 | 3.25067 | 8.05E-05 | 3.50331 |
| 8132960 | SNORA22 | Small nucleolar RNA SNORA22 | 7p11.2 | 0.000176 | 2.57613 | 5.75E-05 | 2.81017 |
| 7950042 | SHANK2 | SH3 and multiple ankyrin repeat domains 2 | 11q13.2 | 0.000183 | 2.57152 | 1.38E-11 | 9.76012 |
| 8016519 | RNU1-42P | RNA, U1 small nuclear 42, pseudogene | 17q21.32 | 0.000191 | 2.634 | 0.003763 | 2.05064 |
| 8030366 | SNORD35A | small nucleolar RNA, C/D box 35A | 19q13.3 | 0.000195 | 3.96719 | 0.000429 | 3.61959 |
| 8151871 | CCNE2 | cyclin E2 | 8q22.1 | 0.000201 | 4.20309 | 5.79E-08 | 11.0599 |
| 8023605 | PIGN | phosphatidylinositol glycan anchor biosynthesis class N | 18q21.33 | 0.000203 | -2.53721 | 0.000297 | -2.46263 |
| 8171876 | METTL1 | methyltransferase like 1 | 12q13 | 0.000206 | 2.20784 | 0.000315 | 2.14566 |
| 8152715 | KLHL38 | kelch-like family member 38 | 8q24.13 | 0.000213 | 2.19691 | 2.81E-05 | 2.509 |
| 7953603 | C1S | complement component 1, s subcomponent | 12p13 | 0.000227 | -3.61763 | 3.21E-05 | -4.46728 |
| 7961418 | RNA5SP353 | RNA, 5S ribosomal pseudogene 353 | 12p13.1 | 0.000229 | 3.49608 | 0.002205 | 2.72879 |
| 8173261 | ZC4H2 | zinc finger, C4H2 domain containing | Xq11.2 | 0.00023 | 2.66397 | 0.000374 | -2.5572 |
| 8092640 | RFC4 | replication factor C (activator 1) 4, 37kDa | 3q27 | 0.000239 | 2.86167 | 0.00134 | 2.44352 |
| 7931108 | DMBT1 | deleted in malignant brain tumors 1 | 10q26.13 | 0.000241 | 2.50455 | 2.36E-05 | 2.99712 |
| 8138799 | TRIL | TLR4 interactor with leucine-rich repeats | 7p14.3 | 0.000243 | 2.34188 | 3.37E-09 | 5.26128 |
| 8086517 | CDCP1 | CUB domain containing protein 1 | 3p21.31 | 0.000246 | 2.32857 | 6.40E-12 | 8.63609 |
| 7943442 | DYNC2H1 | dynein, cytoplasmic 2, heavy chain 1 | 11q21.3 | 0.000246 | -2.88399 | 2.08E-05 | -3.59515 |
| 7938035 | TRIM22 | tripartite motif containing 22 | 11p15 | 0.000252 | -3.12632 | 2.20E-05 | -3.95281 |
| 8126382 | C6orf132 | chromosome 6 open reading frame 132 | 6p21.1 | 0.000253 | 2.41263 | 1.32E-10 | 7.30906 |
| 7898057 | PDPN | podoplanin | 1p36.21 | 0.000267 | -2.47662 | 2.04E-05 | -3.01862 |
| 8124510 | HIST1H2BL | histone cluster 1, H2bl | 6p22.1 | 0.000268 | 2.03149 | 0.000302 | 2.01663 |
| 8009844 | LLGL2 | lethal giant larvae homolog 2 (Drosophila) | 17q25.1 | 0.000275 | 2.1597 | 1.64E-10 | 5.67011 |
| 8038326 | SLC6A16 | solute carrier family 6, member 16 | 19q13.33 | 0.00028 | 2.44241 | 3.02E-05 | 2.895 |
| 7984016 | C2CD4A | C2 calcium-dependent domain containing 4A | 15q22.2 | 0.000297 | 3.22842 | 5.79E-05 | 3.80919 |
| 7925876 | PFKP | phosphofructokinase, platelet | 10p15.2 | 0.000302 | 3.34521 | 0.000819 | 3.00688 |
| 8123864 | TFAP2A | transcription factor AP-2 alpha | 6p24.3 | 0.000328 | 3.41027 | 1.62E-12 | 29.5944 |
| 7990564 |  |  | --- | 0.00033 | 3.94888 | 0.002307 | 3.09907 |
| 8103932 | CENPU | centromere protein U | 4q35.1 | 0.000338 | 2.84849 | 3.58E-08 | 6.53753 |
| 7920928 | PAQR6 | progestin and adipoQ receptor family member VI | 1q22 | 0.000343 | 2.07455 | 1.54E-05 | 2.52583 |
| 7950062 |  |  | --- | 0.000387 | 4.07671 | 0.001099 | 3.56667 |
| 8023882 | ZNF516 | zinc finger protein 516 | 18q23 | 0.000388 | -2.11218 | 0.000169 | -2.23268 |
| 8006865 | PPP1R1B | protein phosphatase 1 regulatory inhibitor subunit 1B | 17q12 | 0.000403 | 4.98582 | 1.93E-05 | 7.67722 |
| 7920252 | S100A7 | S100 calcium binding protein A7 | 1q21 | 0.000406 | 5.56357 | 6.47E-06 | 10.3976 |
| 8056426 | TTC21B | tetratricopeptide repeat domain 21B | 2q24.3 | 0.00041 | -2.0023 | 0.000105 | -2.17904 |
| 7926383 | ENSG00000251803 | Y_RNA ENSG00000251803 | 10p12.33 | 0.000419 | 2.69044 | 6.77E-07 | 4.71046 |
| 7916592 | MYSM1 | Myb-like, SWIRM and MPN domains 1 | 1p32.1 | 0.000486 | 2.20395 | 0.000154 | 2.3941 |
| 7914075 | FCN3 | ficolin 3 | 1p36.11 | 0.000506 | 3.111 | 0.004961 | 2.4242 |
| 8069174 | FAM207A | family with sequence similarity 207, member A | 21q22.3 | 0.000509 | 2.02997 | 0.0001 | 2.25489 |
| 8044700 | DPP10 | dipeptidyl peptidase like 10 | 2q14.1 | 0.000524 | 2.11674 | 0.000144 | 2.31374 |
| 7952341 | CLMP | CXADR-like membrane protein | 11q24.1 | 0.00053 | -3.52565 | 3.43E-07 | -8.11144 |
| 8144036 | XRCC2 | X-ray repair cross complementing 2 | 7q36.1 | 0.000542 | 3.38582 | 4.75E-06 | 5.71114 |
| 8037170 | PAFAH1B3 | platelet activating factor acetylhydrolase 1b catalytic subunit 3 | 19q13.2 | 0.000544 | 2.28355 | 9.60E-05 | 2.6051 |
| 8050702 | TP53I3 | tumor protein p53 inducible protein 3 | 2p23.3 | 0.000558 | -2.36647 | 0.000495 | -2.38948 |
| 7913566 | HTR1D | 5-hydroxytryptamine receptor 1D | 1p36.12 | 0.000655 | 2.3979 | 0.004322 | 2.03704 |
| 8018305 | HN1 | hematological and neurological expressed 1 | 17q25.1 | 0.000678 | 2.42335 | 1.09E-06 | 4.10144 |
| 8158059 | STXBP1 | syntaxin binding protein 1 | 9q34.1 | 0.000693 | 2.25433 | 0.000492 | -2.31524 |
| 8027354 | RPSAP58 | ribosomal protein SA pseudogene 58 | 19p12 | 0.000701 | 2.24406 | 1.96E-05 | 2.94018 |
| 8040985 | ZNF512 | zinc finger protein 512 | 2p23 | 0.000702 | -2.04725 | 0.000293 | -2.17341 |
| 8054702 | CKAP2L | cytoskeleton associated protein 2-like | 2q13 | 0.000715 | 3.46841 | 4.25E-05 | 4.82604 |
| 8131326 | SLC29A4 | solute carrier family 29 (equilibrative nucleoside transport | 7p22.1 | 0.000729 | 2.49447 | 0.000633 | 2.52579 |
| 7954196 | MGST1 | microsomal glutathione S-transferase 1 | 12p12.3 | 0.000736 | 6.35258 | 1.95E-07 | 26.2283 |
| 8147697 | GRHL2 | grainyhead-like 2 (Drosophila) | 8q22.3 | 0.000757 | 2.08184 | 8.52E-19 | 39.8456 |
| 8047487 | FZD7 | frizzled class receptor 7 | 2q33 | 0.0008 | -4.55755 | 1.86E-05 | -7.82385 |
| 7985873 | TICRR | TOPBP1-interacting checkpoint and replication regulator | 15q26.1 | 0.00081 | 3.8864 | 8.97E-07 | 9.26681 |
| 8105434 | RNU6-299P | RNA, U6 small nuclear 299, pseudogene | 5q11.2 | 0.000825 | 2.47507 | 0.001444 | 2.35351 |
| 8104680 | C5orf22 | chromosome 5 open reading frame 22 | 5p13.3 | 0.000829 | 2.22846 | 0.0021 | 2.06837 |
| 8147206 | RIPK2 | receptor-interacting serine-threonine kinase 2 | 8q21 | 0.000831 | 2.26532 | 0.000214 | 2.52236 |
| 7974229 | KLHDC2 | kelch domain containing 2 | 14q21.3 | 0.000873 | -2.18057 | 0.001562 | -2.08369 |
| 8180255 | HIST2H4A | histone cluster 2 H4 family member a | 1q21.2 | 0.000874 | 3.65095 | 0.000462 | 3.96039 |
| 8180321 | HIST2H4A | histone cluster 2 H4 family member a | 1q21.2 | 0.000874 | 3.65095 | 0.000462 | 3.96039 |
| 8085754 | SGOL1 | shugoshin-like 1 (S. pombe) | 3p24.3 | 0.000878 | 4.13578 | 4.45E-06 | 8.44438 |
| 7917976 | SASS6 | spindle assembly 6 homolog (C. elegans) | 1p21.2 | 0.000904 | 2.25693 | 0.000177 | 2.56907 |
| 8143772 | RARRES2 | retinoic acid receptor responder 2 | 7q36 | 0.000944 | -4.88626 | 0.000175 | -6.35356 |
| 7937079 | BNIP3 | BCL2 adenovirus E1B 19kDa interacting protein 3 | 10q26.3 | 0.000945 | 2.76083 | 0.000202 | 3.22 |
| 7950307 | UCP2 | uncoupling protein 2 (mitochondrial, proton carrier) | 11q13 | 0.000949 | 2.50606 | 0.00336 | 2.22457 |
| 8068570 | DSCR8 | Down syndrome critical region gene 8 | 21q22.2 | 0.000952 | 2.29781 | 0.000259 | 2.55706 |
| 7983306 | WDR76 | WD repeat domain 76 | 15q15.3 | 0.000962 | 2.63842 | 0.000402 | 2.86944 |
| 8107532 | HSD17B4 | hydroxysteroid (17-beta) dehydrogenase 4 | 5q21 | 0.000978 | -2.28875 | 0.000721 | -2.34744 |
| 8144742 | MICU3 | mitochondrial calcium uptake family, member 3 | 8p22 | 0.001006 | -2.18177 | 2.18E-07 | -4.11032 |
| 7985213 | CHRNA5 | cholinergic receptor, nicotinic, alpha 5 (neuronal) | 15q24 | 0.001018 | 4.40657 | 2.60E-06 | 10.3586 |
| 7919612 | HIST2H3A | histone cluster 2, H3a | 1q21.2 | 0.001037 | 2.87055 | 2.72E-05 | 4.17643 |
| 7970513 | SKA3 | spindle and kinetochore associated complex subunit 3 | 13q12.1 | 0.001043 | 3.38231 | 5.00E-06 | 6.35801 |
| 8043602 | NCAPH | non-SMC condensin I complex, subunit H | 2q11.2 | 0.001083 | 2.93165 | 2.07E-06 | 5.63973 |
| 8096160 | ARHGAP24 | Rho GTPase activating protein 24 | 4q22.1 | 0.001087 | -2.28822 | 3.50E-06 | -3.6325 |
| 8123598 | SERPINB1 | serpin family B member 1 | 6p25.2 | 0.001089 | -2.0875 | 1.50E-05 | -2.84213 |
| 8050350 | RNA5SP85 | RNA, 5S ribosomal pseudogene 85 | 2p25.1 | 0.0011 | 4.16738 | 0.001554 | 3.96124 |
| 8094501 | STIM2 | stromal interaction molecule 2 | 4p15.2 | 0.001103 | -2.10354 | 1.12E-06 | -3.46346 |
| 8131179 | TTYH3 | tweety family member 3 | 7p22 | 0.001109 | 2.61068 | 4.90E-06 | 4.339 |
| 8096314 | PKD2 | polycystic kidney disease 2 (autosomal dominant) | 4q22.1 | 0.001147 | -2.25476 | 4.72E-05 | -2.91624 |
| 8046333 | CYBRD1 | cytochrome b reductase 1 | 2q31.1 | 0.001162 | -2.72588 | 1.47E-07 | -6.57034 |
| 8033054 | FUT3 | fucosyltransferase 3 (Lewis blood group) | 19p13.3 | 0.001181 | 3.20483 | 2.76E-06 | 6.40595 |
| 8062461 | LBP | lipopolysaccharide binding protein | 20q11.23 | 0.001182 | 8.05606 | 8.96E-05 | 13.8036 |
| 8109697 | CCNG1 | cyclin G1 | 5q34 | 0.001194 | 2.47625 | 6.70E-05 | -3.21448 |
| 7982792 | RAD51 | RAD51 recombinase | 15q15.1 | 0.001194 | 2.4666 | 1.61E-06 | 4.431 |
| 8026398 | CASP14 | caspase 14, apoptosis-related cysteine peptidase | 19p13.1 | 0.00122 | 11.7123 | 8.28E-06 | 39.4579 |
| 8058127 | CLK1 | CDC-like kinase 1 | 2q33 | 0.001223 | -2.43026 | 0.00332 | -2.21281 |
| 8073062 | APOBEC3B | apolipoprotein B mRNA editing enzyme catalytic subunit 3B | 22q13.1 | 0.001257 | 3.52411 | 0.000299 | 4.23826 |
| 8092457 | ALG3 | ALG3, alpha-1,3- mannosyltransferase | 3q27.1 | 0.001297 | 2.1047 | 0.001488 | 2.08229 |
| 8016033 | FAM171A2 | family with sequence similarity 171, member A2 | 17q21.31 | 0.001324 | 2.11721 | 0.000184 | -2.46261 |
| 8113050 | CETN3 | centrin, EF-hand protein, 3 | 5q14.3 | 0.001339 | -2.28378 | 0.003618 | -2.09155 |
| 8006984 | PSMD3 | proteasome 26S subunit, non-ATPase 3 | 17q | 0.001361 | 2.81628 | 8.22E-05 | 3.78366 |
| 8132318 | ANLN | anillin, actin binding protein | 7p14.2 | 0.001361 | 3.16922 | 4.57E-08 | 10.3536 |
| 8114797 | SPRY4 | sprouty homolog 4 (Drosophila) | 5q31.3 | 0.001367 | 3.00741 | 0.00066 | 3.26948 |
| 7990774 | RASGRF1 | Ras protein-specific guanine nucleotide-releasing factor 1 | 15q25.1 | 0.00143 | 2.52753 | 1.26E-05 | 3.93562 |
| 7946988 | HPS5 | Hermansky-Pudlak syndrome 5 | 11p14 | 0.001471 | -2.28995 | 0.00023 | -2.68596 |
| 7905147 | C1orf54 | chromosome 1 open reading frame 54 | 1q21.2 | 0.001478 | -2.11752 | 4.93E-08 | -4.60829 |
| 7954559 | PPFIBP1 | PPFIA binding protein 1 | 12p11.23p22 | 0.001503 | -2.38134 | 6.08E-06 | -3.86247 |
| 8154178 | JAK2 | Janus kinase 2 | 9p24 | 0.001546 | -2.27015 | 8.72E-06 | -3.49504 |
| 7954481 | SSPN | sarcospan | 12p11.2 | 0.001639 | -2.76238 | 0.001098 | -2.88578 |
| 7980861 | CATSPERB | catsper channel auxiliary subunit beta | 14q32.12 | 0.001697 | 3.89937 | 3.31E-08 | 17.6191 |
| 8128123 | RRAGD | Ras-related GTP binding D | 6q15 | 0.001698 | 3.14322 | 1.60E-05 | 5.45213 |
| 8029465 | BCL3 | B-cell CLL/lymphoma 3 | 19q13.32 | 0.001772 | 2.18742 | 1.96E-05 | 3.15386 |
| 8116734 | LY86 | lymphocyte antigen 86 | 6p25.1 | 0.001902 | -3.09987 | 0.000858 | -3.41962 |
| 8120838 | TTK | TTK protein kinase | 6q14.1 | 0.001946 | 3.21036 | 5.50E-08 | 11.4048 |
| 7919151 | RNVU1-10 | RNA, variant U1 small nuclear 10 | 1q21.2 | 0.001959 | 2.88312 | 0.004183 | 2.63338 |
| 7919392 | RNVU1-10 | RNA, variant U1 small nuclear 10 | 1q21.2 | 0.001959 | 2.88312 | 0.004183 | 2.63338 |
| 7910030 | DNAH14 | dynein, axonemal, heavy chain 14 | 1q42.12 | 0.001968 | 2.58835 | 0.006057 | 2.29157 |
| 8117301 | HIST1H2BA | histone cluster 1, H2ba | 6p22.2 | 0.001993 | 2.38777 | 0.001702 | 2.42444 |
| 7958019 | DRAM1 | DNA-damage regulated autophagy modulator 1 | 12q23.2 | 0.001999 | -2.25186 | 0.006502 | -2.01831 |
| 8121749 | GJA1 | gap junction protein, alpha 1, 43kDa | 6q22.31 | 0.002032 | -5.06345 | 0.00022 | -7.46954 |
| 7924910 | ACTA1 | actin, alpha 1, skeletal muscle | 1q42.13 | 0.002034 | 2.51037 | 0.000398 | 2.95542 |
| 8021169 | LIPG | lipase, endothelial | 18q21.1 | 0.002043 | 2.63952 | 0.000205 | 3.35712 |
| 8148559 | THEM6 | thioesterase superfamily member 6 | 8q24.3 | 0.002062 | 2.3231 | 0.000375 | 2.71572 |
| 8103326 | FGG | fibrinogen gamma chain | 4q28 | 0.002109 | 3.05913 | 0.001045 | 3.33734 |
| 7902861 | LRRC8B | leucine rich repeat containing 8 family, member B | 1p22.2 | 0.002166 | 2.30421 | 4.99E-06 | 3.93596 |
| 8034202 | RAB3D | RAB3D, member RAS oncogene family | 19p13.2 | 0.002249 | 2.09335 | 0.000763 | 2.28777 |
| 7915612 | PTCH2 | patched 2 | 1p34.1 | 0.002252 | 5.29555 | 0.000113 | 9.09492 |
| 8094759 | NSUN7 | NOP2/Sun RNA methyltransferase family member 7 | 4p14 | 0.002257 | 2.0687 | 8.34E-05 | 2.68123 |
| 8003667 | SERPINF1 | serpin family F member 1 | 17p13.3 | 0.002346 | -4.72701 | 0.000509 | -6.15215 |
| 8121405 | LOC100996634 | transmembrane protein FLJ37396 | 6q21 | 0.002347 | 2.18544 | 0.001862 | 2.23082 |
| 7954469 | RASSF8 | Ras association domain family member 8 | 12p12.1 | 0.002384 | -2.45447 | 1.57E-06 | -4.94581 |
| 8146921 | RDH10 | retinol dehydrogenase 10 (all-trans) | 8q21.11 | 0.002492 | 4.12628 | 0.002013 | 4.27182 |
| 7993296 | SHISA9 | shisa family member 9 | 16p13.12 | 0.002539 | 2.70471 | 0.000779 | 3.08915 |
| 8043835 | C2orf15 | chromosome 2 open reading frame 15 | 2q11.2 | 0.002558 | 2.54117 | 0.000184 | 3.33585 |
| 8104131 | MGC39584 | uncharacterized LOC441058 | 21p11.2 | 0.002565 | 2.27192 | 0.000217 | 2.84629 |
| 8105409 | RNA5SP183 | RNA, 5S ribosomal pseudogene 183 | 5q11.2 | 0.002567 | 2.14995 | 0.004833 | 2.03106 |
| 7917199 | TTLL7 | tubulin tyrosine ligase-like family, member 7 | 1p31.1 | 0.00257 | 2.71046 | 2.45E-06 | 5.71754 |
| 8036473 | PPP1R14A | protein phosphatase 1, regulatory (inhibitor) subunit 14A | 19q13.2 | 0.002651 | 2.24546 | 0.001846 | 2.32268 |
| 8017262 | BRIP1 | BRCA1 interacting protein C-terminal helicase 1 | 17q22.2 | 0.002677 | 3.76849 | 2.60E-06 | 10.2017 |
| 7910674 | RNU6-968P | RNA, U6 small nuclear 968, pseudogene | 1q42.3 | 0.002724 | -2.49734 | 0.000155 | -3.34601 |
| 7965541 | FGD6 | FYVE, RhoGEF and PH domain containing 6 | 12q22 | 0.002794 | -2.19748 | 2.50E-06 | -4.00802 |
| 8020630 | TTC39C | tetratricopeptide repeat domain 39C | 18q11.2 | 0.002841 | -2.15753 | 0.000318 | 2.61086 |
| 7986068 | BLM | Bloom syndrome, RecQ helicase-like | 15q26.1 | 0.002848 | 2.87874 | 3.09E-06 | 6.32783 |
| 7905576 | S100A7A | S100 calcium binding protein A7A | 1q21.3 | 0.002871 | 3.13397 | 0.000216 | 4.37528 |
| 7930380 | ADD3 | adducin 3 (gamma) | 10q25.2 | 0.002886 | -2.21965 | 4.25E-10 | -8.88016 |
| 8060344 | TRIB3 | tribbles pseudokinase 3 | 20p13 | 0.002912 | 2.02098 | 8.06E-08 | 4.51698 |
| 8008372 | EPN3 | epsin 3 | 17q21.33 | 0.003127 | 2.10327 | 1.76E-06 | 3.88446 |
| 8144802 | PDGFRL | platelet-derived growth factor receptor-like | 8p22 | 0.003135 | -5.13015 | 0.001444 | -5.95487 |
| 8054580 | BUB1 | BUB1 mitotic checkpoint serine/threonine kinase | 2q14 | 0.003286 | 3.35553 | 6.85E-06 | 7.72485 |
| 7916167 | ORC1 | origin recognition complex, subunit 1 | 1p32 | 0.003358 | 2.37857 | 4.53E-06 | 4.50858 |
| 8101701 | PPM1K | protein phosphatase, Mg2+/Mn2+ dependent 1K | 4q22.1 | 0.003393 | -2.57908 | 0.004487 | -2.49709 |
| 8144758 | ZDHHC2 | zinc finger, DHHC-type containing 2 | 8p22 | 0.003403 | -2.44174 | 0.000133 | -3.40382 |
| 8018352 | SLC25A19 | solute carrier family 25 member 19 | 17q25.1 | 0.003498 | 2.20717 | 0.004391 | 2.15905 |
| 8147373 | RP11-22C11.1 | RP11-22C11.1 ENSG00000241003 | 8q22.1 | 0.003532 | 2.31258 | 2.53E-09 | 8.78401 |
| 8174610 | LRCH2 | leucine rich repeats and calponin homology domain containing 2 | Xq23 | 0.003546 | -2.26716 | 2.64E-05 | -3.57769 |
| 8174092 | ARMCX2 | armadillo repeat containing, X-linked 2 | Xq22.1 | 0.00356 | -2.4408 | 6.41E-06 | -4.60954 |
| 8096176 | PTPN13 | protein tyrosine phosphatase, non-receptor type 13 | 4q21.3 | 0.003689 | -2.37306 | 2.23E-10 | -12.2684 |
| 7968212 | WASF3 | WAS protein family, member 3 | 13q12 | 0.003693 | 2.23064 | 0.000583 | -2.65836 |
| 8138997 | TBX20 | T-box 20 | 7p14.3 | 0.003785 | 2.03991 | 0.000326 | 2.50635 |
| 7952673 | FLJ45950 | FLJ45950 protein | 11q24.3 | 0.003796 | 2.50132 | 0.000138 | 3.56485 |
| 8033767 | ZNF560 | zinc finger protein 560 | 19p13.2 | 0.00389 | 2.54178 | 0.006311 | 2.40171 |
| 8014903 | GSDMB | gasdermin B | 17q12 | 0.004139 | 3.2994 | 6.25E-05 | 5.92759 |
| 8124604 |  |  | --- | 0.004247 | 2.13784 | 0.000617 | 2.55258 |
| 7982757 | CASC5 | cancer susceptibility candidate 5 | 15q14 | 0.004301 | 2.43946 | 2.32E-05 | 4.20052 |
| 8079237 | KIF15 | kinesin family member 15 | 3p21.31 | 0.004392 | 2.60961 | 0.000176 | 3.77281 |
| 7944554 | TMEM136 | transmembrane protein 136 | 11q23.3 | 0.004483 | -2.23737 | 0.003503 | -2.29428 |
| 8166730 | CYBB | cytochrome b-245, beta polypeptide | Xp21.1 | 0.004533 | -2.71703 | 0.000118 | -4.20155 |
| 7907297 | FMO4 | flavin containing monooxygenase 4 | 1q24.3 | 0.004561 | -2.68904 | 0.00032 | -3.69681 |
| 8131263 | SNORD13P2 | small nucleolar RNA, C/D box 13 pseudogene 2 | 7p22.1 | 0.004703 | 2.27431 | 0.004612 | 2.27897 |
| 7909503 | SERTAD4 | SERTA domain containing 4 | 1q32.2 | 0.004703 | 2.94704 | 0.000489 | 3.9762 |
| 7982889 | NUSAP1 | nucleolar and spindle associated protein 1 | 15q15.1 | 0.004721 | 2.97978 | 0.000157 | 4.66155 |
| 8134631 | ZNF655 | zinc finger protein 655 | 7q22.1 | 0.004764 | -2.20819 | 0.000417 | -2.79628 |
| 7951077 | SESN3 | sestrin 3 | 11q21 | 0.004916 | -2.93392 | 2.37E-06 | -7.69952 |
| 7950641 | NDUFC2-KCTD14 | NDUFC2-KCTD14 readthrough | 11q14.1 | 0.004975 | -3.01364 | 0.000484 | -4.13902 |
| 8112668 | GCNT4 | glucosaminyl (N-acetyl) transferase 4, core 2 | 5q12 | 0.005152 | -2.9007 | 0.005615 | -2.86618 |
| 8135915 | HILPDA | hypoxia inducible lipid droplet-associated | 7q32.1 | 0.005592 | 2.12064 | 1.82E-06 | 4.36251 |
| 8049317 | DGKD | diacylglycerol kinase delta | 2q37.1 | 0.005674 | 2.24591 | 0.005683 | 2.24556 |
| 8150002 | FBXO16 | F-box protein 16 | 8p21.1 | 0.005732 | 2.02207 | 0.005676 | 2.02392 |
| 7937772 |  |  | --- | 0.005803 | 2.93767 | 2.93E-05 | -5.89363 |
| 8071212 | CDC45 | cell division cycle 45 | 22q11.21 | 0.005909 | 2.40018 | 2.12E-06 | 5.54402 |
| 7953943 | GABARAPL1 | GABA(A) receptor-associated protein like 1 | 12p13.2 | 0.005952 | 2.06463 | 5.85E-05 | -3.12222 |
| 8112327 | CKS1B | CDC28 protein kinase regulatory subunit 1B | 1q21.2 | 0.005966 | 2.33536 | 0.000288 | 3.22769 |
| 7952526 | CDON | cell adhesion associated, oncogene regulated | 11q24.2 | 0.006017 | -2.83983 | 9.46E-07 | -8.54508 |
| 8096704 | NPNT | nephronectin | 4q24 | 0.006183 | -3.86649 | 6.04E-09 | -37.242 |
| 8069700 | N6AMT1 | N-6 adenine-specific DNA methyltransferase 1 (putative) | 21q21 | 0.006198 | -2.0165 | 0.001055 | -2.3682 |
| 8006958 | ZPBP2 | zona pellucida binding protein 2 | 17q12 | 0.006363 | 3.1968 | 0.001034 | 4.20632 |
| 7939383 | PRR5L | proline rich 5 like | 11p13 | 0.006429 | -2.24782 | 0.00185 | -2.56727 |
| 8057394 | SESTD1 | SEC14 and spectrin domains 1 | 2q31.2 | 0.006465 | -2.07068 | 0.001085 | -2.45288 |
| 7926896 | CKS1B | CDC28 protein kinase regulatory subunit 1B | 1q21.2 | 0.006622 | 2.27951 | 0.000325 | 3.13377 |
| 7913385 | RAP1GAP | RAP1 GTPase activating protein | 1p36.12 | 0.006943 | 2.04769 | 0.000179 | 2.86462 |
| 8109926 | GABRP | gamma-aminobutyric acid type A receptor pi subunit | 5q35.1 | 0.006943 | 7.11888 | 7.75E-07 | 64.1157 |
| 8051298 | GALNT14 | polypeptide N-acetylgalactosaminyltransferase 14 | 2p23.1 | 0.006947 | 2.14477 | 5.88E-05 | 3.39854 |
| 8133233 | AUTS2 | autism susceptibility candidate 2 | 7q11.22 | 0.006968 | 2.23131 | 0.005166 | 2.3059 |
| 7916898 | DEPDC1 | DEP domain containing 1 | 1p31.2 | 0.006971 | 3.06839 | 1.30E-06 | 10.0545 |
| 8012949 | FBXW10 | F-box and WD repeat domain containing 10 | 17p12 | 0.007177 | 2.1685 | 3.20E-05 | 3.68095 |
| 8120335 | FAM83B | family with sequence similarity 83, member B | 6p12.1 | 0.007264 | 3.6984 | 6.58E-08 | 24.1385 |
| 8000480 | RNU6-1241P | RNA, U6 small nuclear 1241, pseudogene | 16p12.1 | 0.007271 | 2.61971 | 0.000171 | -4.17481 |
| 7988644 | ATP8B4 | ATPase, class I, type 8B, member 4 | 15q21.2 | 0.00739 | -2.15857 | 8.16E-06 | -4.16721 |
