# Supplementary figures and images for: Comprehensive molecular biomarker identification in breast cancer brain metastases
Source: J Transl Med. 2017 Dec 29;15:269. doi: 10.1186/s12967-017-1370-x (PMC5747948; doi:10.1186/s12967-017-1370-x)

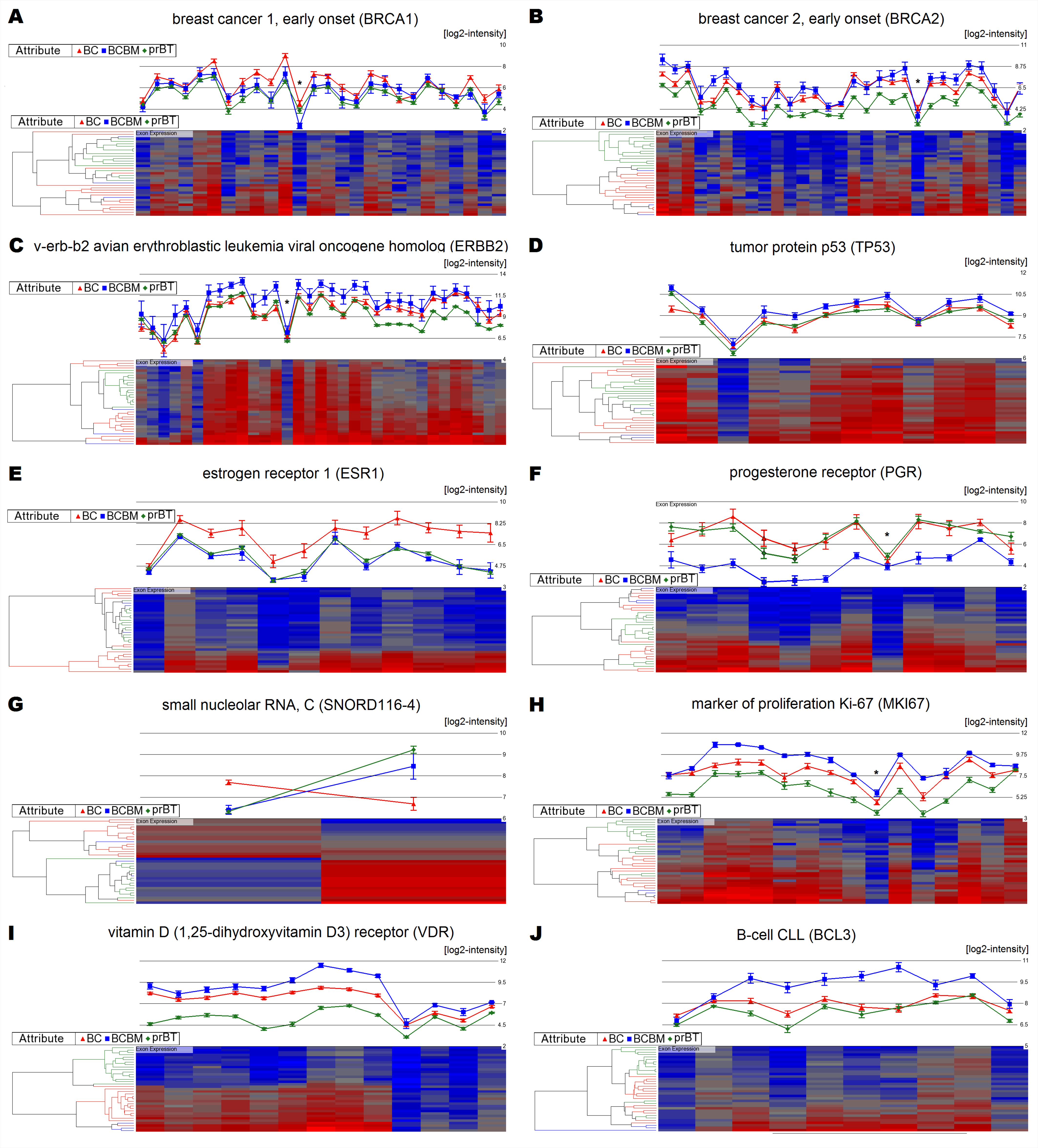

Supplement: Supplementary file 2 — Additional file 2. Exon expression levels for a number of cancer associated genes. A, BRCA1; B, BRCA2; C, ERBB2; D, TP53; E, ER1 (ESR1); F, PR (PGR); G, SNORD116-4; H, MKI67; I, VDR; and J, BCL3. Comparably low expression of a number of exon probes can be presumbably attributed to splicing events of transcripts; asterisks mark examples. BCL3 is upregulated on the gene expression level (Additional file 1). Notably, 5` located exons of ERBB2 are lower expressed than 3` located exons in prBT and expression levels of the two probes covering the SNORD116-4 transcript diverge in BCBM and prBT compared to BC. Exon probes are displayed from 5` (left) to 3` (right) of the transcripts. Blue and red colors in heat maps refer to lower and higher expression, respectively. [file 12967_2017_1370_MOESM2_ESM.tif]
